# Web-based Cognitive Behavior Therapy: Analysis of Site Usage and Changes in Depression and Anxiety Scores

**DOI:** 10.2196/jmir.4.1.e3

**Published:** 2002-02-15

**Authors:** Helen Christensen, Kathleen M Griffiths, Ailsa Korten

**Affiliations:** ^1^The Centre for Mental Health ResearchThe Australian National UniversityCanberraAustralia

**Keywords:** Internet, depression, primary prevention, program evaluation

## Abstract

**Background:**

Cognitive behavior therapy is well recognized as an effective treatment and prevention for depression when delivered face-to-face, via self-help books (bibliotherapy), and through computer administration. The public health impact of cognitive behavior therapy has been limited by cost and the lack of trained practitioners. We have developed a free Internet-based cognitive behavior therapy intervention (MoodGYM, http://moodgym.anu.edu.au) designed to treat and prevent depression in young people, available to all Internet users, and targeted to those who may have no formal contact with professional help services.

**Objective:**

To document site usage, visitor characteristics, and changes in depression and anxiety symptoms among users of MoodGYM, a Web site delivering a cognitive-behavioral-based preventive intervention to the general public.

**Methods:**

All visitors to the MoodGYM site over about 6 months were investigated, including 2909 registrants of whom 1503 had completed at least one online assessment. Outcomes for 71 university students enrolled in an Abnormal Psychology course who visited the site for educational training were included and examined separately. The main outcome measures were (1) site-usage measures including number of sessions, hits and average time on the server, and number of page views; (2) visitor characteristics including age, gender, and initial Goldberg self-report anxiety and depression scores; and (3) symptom change measures based on Goldberg anxiety and depression scores recorded on up to 5 separate occasions.

**Results:**

Over the first almost-6-month period of operation, the server recorded 817284 hits and 17646 separate sessions. Approximately 20% of sessions lasted more than 16 minutes. Registrants who completed at least one assessment reported initial symptoms of depression and anxiety that exceeded those found in population-based surveys and those characterizing a sample of University students. For the Web-based population, both anxiety and depression scores decreased significantly as individuals progressed through the modules.

**Conclusions:**

Web sites are a practical and promising means of delivering cognitive behavioral interventions for preventing depression and anxiety to the general public. However, randomized controlled trials are required to establish the effectiveness of these interventions.

## Introduction

It is well recognized that cognitive behavior therapy (CBT) is an effective treatment for depression when delivered face-to-face, via self-help books (bibliotherapy), and through computer administration [1,2,3]. CBT programs have also been shown to be effective in preventing depression [4,5,6]. However, the public health impact of these treatments and programs has been limited by cost and the lack of trained practitioners and programs.

MoodGYM is a free Internet-based CBT intervention designed to treat and prevent depression in young people with access to the Internet (for screenshots see PowerPoint Multimedia Appendix). Where face-to-face treatment or prevention using CBT is unavailable, the Internet provides an excellent way of disseminating preventive CBT programs. The information is widely accessible, can be updated, is available 24 hours a day, and is self-paced. The interactive and multimedia possibilities afforded by standard Web browsers offer the potential to engage the target population in ways that are not possible using conventional delivery methods. The Internet is able to support software applications that can be tailored to individual needs, and such customized interventions are recognized as important ingredients in successful prevention work [7].

To date, mental health Web sites have been used to provide information [8], to survey mental health [9], to assist in the delivery of anxiety treatment [10], and to provide support [11]. However, they have not been widely used to deliver specific mental health prevention interventions to all Internet users.

We describe the usage of the MoodGYM site and the characteristics and outcomes of the first visitors and registrants to the site over almost a 6-month period. In this paper, we report on 3 aspects:

1. site usage information, including the number of users who register on the site, the number of sessions recorded, the dates and times when modules were completed, and average time on the site;
2. characteristics of registrants including gender, age, and scores on the Goldberg Anxiety and Depression Scales [12];
3. change in anxiety and depression scores experienced by registrants as they progress through the site (because the assessments are repeated, we were able to examine whether psychological distress decreases as a function of module use).

## Methods

### Participants

Data from all visitors were recorded in the almost-6-month period between the release of the site on April 1, 2001 to the download of data on September 27, 2001. Visitors were individuals who accessed at least one page of the site. Registrants were individuals who entered details about themselves on the site, gave permission for their data to be used in research, and were allocated an individual database record. Registration was required before participants were able to access the site modules. There were 2909 registrants. Of these, 1503 completed one or more online assessments. Also, 71 university students enrolled in an Abnormal Psychology course who visited the site for educational training were included and examined separately. The students gave permission for their server data to be used for research purposes although they were not explicitly aware that their data would be compared directly with data of general public users.

### Site Description

The site consists of a set of 5 cognitive behavioral training modules, a personal workbook (containing 29 exercises and assessments) that records and updates each user's responses, an interactive game, and a feedback evaluation form. Module 1 introduces the site "characters" (who model patterns of dysfunctional thinking) and demonstrates the way in which mood is influenced by thinking, using animated diagrams and interactive exercises. Module 2 describes types of dysfunctional thinking, the methods to overcome them, and provides self-assessment of "warpy" (dysfunctional) thoughts. Module 3 provides behavioral methods to overcome dysfunctional thinking, and includes sections on assertiveness and self-esteem training. Module 4 assesses life-event stress, pleasant events, and activities, and provides 3 downloadable relaxation tapes. Module 5 covers simple problem solving and typical responses to relationship breakup. Workbook exercises are integrated seamlessly into each of the modules.

Each module was designed to take from 30 minutes to 45 minutes to complete, but users can opt to skip sections. Module 1 has approximately 30 "pages" but many of these contain browser-supported interactive features (creating additional pages) and supplementary pop-up windows. Module 3 has over 60 pages, but users are directed to specific sections depending on their scores on earlier tests and thus may not access all pages.

Online assessments include the Goldberg Depression and Anxiety Scales [12]. Each of the Goldberg scales comprises 9 items. These scales are ideal for use on the Internet because they are brief, well accepted, of satisfactory reliability and validity, have been previously used in epidemiological survey research using a handheld computer interface [13], and their use on our site does not breach copyright. The scales are administered prior to each module.

Although users were encouraged to proceed through the assessments and modules in order, they were free to move about within the site at will. Thus, some registrants started with later modules and did not necessarily work through them in order. Data from each registrant was recorded in an SQL (Structured Query Language) database on a stand-alone server.

### Web-data Retrieval

Server Web statistics were processed using LiveStats [14] and a computer program tailor written for the current analysis.

## Results

### Site Usage Statistics

A total of 17646 sessions were recorded from April 1, 2001, through September 27, 2001. Sessions provide an indication of the number of visitors to the site. Since visitors can access the site more than once, the number of sessions is a good but imperfect indicator of the number of visitors. Across the 181 days, the site recorded 817284 hits and 297046 page views. A hit is an initial request to a computer to deliver a file and is a rough indicator of the amount of Web traffic on a site. On average, each session lasted 9.47 minutes. However, many visitors spent less than 1 minute on the site during which time they viewed only 1 or 2 pages. Table 1 shows the breakdown of sessions as a function of the number of pages viewed and the length of time on the site. Approximately 20% of sessions lasted 16 minutes or more, indicating that individuals were interacting with the material for extended periods. Session statistics include return visits so these summary data are likely to underestimate individual exposure time. Web analysis suggests that individuals spend between 0.6 to 6.7 minutes per site on average [15].

**Table 1:** Page views and time spent per session as a function of percentage of sessions at the MoodGYM site

**Page Views Per Session**	**% of Sessions** (n = 17646)
1	45.09
2-5	17.94
6-10	8.44
11-20	6.11
21-50	10.82
51-100	7.62
101 or more	3.80
	
**Time (Minutes) Per Session.**	**% of Sessions** (n = 17646)
1	48.59
2-5	14.65
6-15	16.84
16-30	10.56
31-45	5.11
46-60	2.10
61 or more	1.96

The number of sessions each day across the 181 days varied from 34 to 359. For those sessions where the visitor's location could be identified, the most common geographical location of the visitor was the US (34.9%) followed by Australia (33.2%), Asia (6.9%) and Europe (1.3%). Apart from some limited media publicity in Australia in May and July, there was no direct marketing of the site.

The mean and median ages of users who supplied age data was 35.5 (SD = 13.0, range = 10 to 80), and 34 respectively. To enable gender-specific information to be returned to the user, gender was a required field. Sixty percent of users were female.

### Anxiety and Depression Scores at Module 1

Of the 2909 people who registered, 1503 completed at least 1, and 465 at least 2 of the depression assessments. Some registrants chose to start with later assessments, so only 1145 people completed the assessment associated with Module 1. A total of 1049 completed at least 1 and 223 at least 2 of the anxiety assessments although only 717 completed the anxiety scale for Module 1.

Scores for the Goldberg Depression Scale and Goldberg Anxiety Scale at Module 1 as a function of gender are shown in Table 2. Also shown are the scores achieved by a representative population sample of 2354 young adults aged 20-24 from the Canberra region [13]. This sample completed the scales anonymously on hand-held computers, but in the presence of interviewers, as part of a large survey of health and well-being.

**Table 2:** Mean module-1 Goldberg Depression and Anxiety Scale scores, with 95% confidence interval (CI) for the self-selected MoodGYM web-based sample, university students completing MoodGYM as part of their studies, and a population sample of 20-24 year olds who completed the scales as part of a survey of health and well-being

	Score (95% CI)* n†			
	Goldberg Depression Scale		Goldberg Anxiety Scale	
	Males	Females	Males	Females
Web-based sample	4.83 (4.57-5.09) n = 406	5.22 (5.03-5.41) n = 690	5.27 (4.96-5.57) n = 280	5.78 (5.54-6.02) n = 406
University students	3.83 (0.95-6.71) n = 6	2.56 (1.88-3.24) n = 43	1.75 (-0.27-3.77) n = 4	3.89 (3.10-4.68) n = 27
Population survey of 20-24 year olds	2.59 (2.45-2.72) n = 1155	3.18 (3.04-3.32) n = 1230	3.20 (3.05-3.35) n = 1155	4.43 (4.28-4.58) n = 1230
† n indicates number of people.				

Analyses of variance indicate that both depression and anxiety scores are significantly higher for females than for males for the Web-based sample (*P*< .0001 for depression; *P*= .006 for anxiety), that there is no significant difference between the population sample and the sample of university students (*P*= .897 for depression; *P*= .600 for anxiety) but that the Web-based sample has significantly higher scores than either the population sample or the university students (*P*< .0001 for both anxiety and depression; pairwise comparisons using Bonferroni correction) where the critical value ( **a** ) is divided by the number of comparisons (in STATA -7 software [16]). These findings suggest that visitors to the site have much higher levels of anxiety and depression than are present in the Canberra community. The possibility that the higher scores in the registrants result from the use of Web-based questionnaire methods rather than computer administration is unlikely, particularly given that the University student's scores did not differ from the scores of the representative sample.

### Change Scores for Anxiety and Depression

#### First Analysis

Our first analysis assumed that users progressed through the modules in order, but that not all modules were necessarily completed. The analysis included all individuals who had completed at least 2 modules. To predict the depression and anxiety scores, we fitted regression models for repeated-measure data, with random effects for individuals, to the data using the xtreg procedure in STATA-7 software [16]. The xtreg procedure estimates linear regression in panel data where there are complex error structures. It is useful where data are correlated, as in repeated-measures designs. Predictors were gender and module. We made separate analyses for the Web-based population and the university students, because of complex significant interaction terms.

For the Web-based population, both depression and anxiety scores decreased significantly as individuals progressed through the modules. Depression scores decreased significantly with module, (Beta = -0.67; 95% CI = -0.80 to -0.55; *P*< .0001), indicating that depression scores fall on average nearly 3 points (2.7; 95% CI = 2.2 to 4.2) if all 5 modules are completed. Females had significantly higher depression scores than males (Beta = 0.62, 95% CI 0.13,1.11, *P*= .014). Anxiety scores decreased significantly with module, (Beta = -0.82; 95% CI = -1.06 to -0.58; *P*< .0001), indicating a decrease on average of more than 3 points (3.3; 95% CI = 2.3 to 4.2) over the 5 modules. There was no evidence of nonlinearity and there were no significant differences in anxiety scores for males and females (Beta = 0.53, 95% CI=-0.19 to 1.25; *P*= .150). Scores for the group of 71 university students who completed the modules as part of their abnormal psychology course were lower than for the Web-based sample and there was no significant change across the modules.

**Figure 1:**
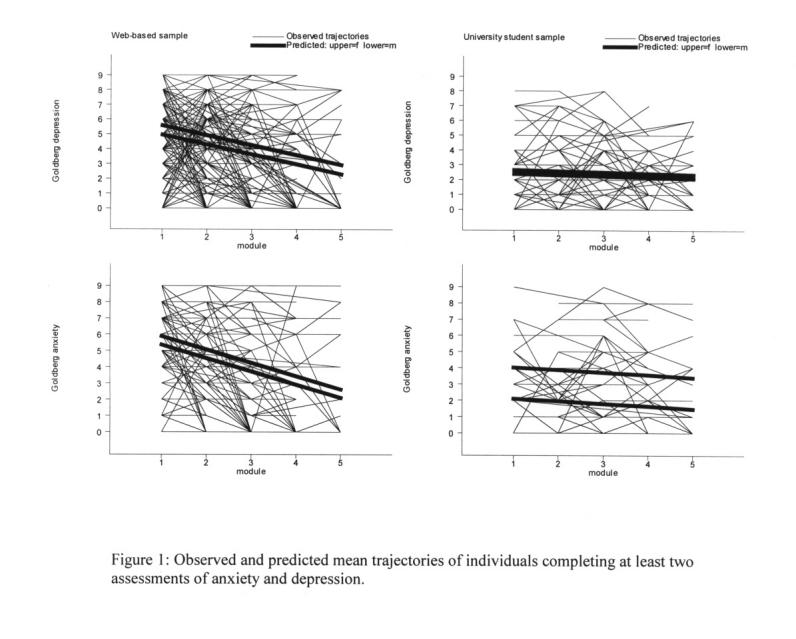
Observed and predicted mean trajectories of individuals completing at least two assessments of anxiety and depression

Figure 1 plots the actual trajectories (paths) of those individuals who completed assessments in depression or anxiety for at least 2 of the modules. Figure 1 also shows, in heavier lines, the predicted trajectories for females (upper line) and males (lower line) based on the statistical modeling described above.

#### Second Analysis

Our second analysis was for individuals with adequate data on the dates and times when modules were completed. The change in scores between the first occasion of measurement and the last occasion, independently of which modules were completed, were compared using repeated-measures ANOVA (Analysis of Variance) from SPSS-10 [17]. Independent variables were gender and time between first and last assessment. Time between assessments was recoded into 3 categories: completed on the same day (n = 869 for depression, n = 644 for anxiety), last assessment completed within one week of the first (n = 31 for depression, n = 18 for anxiety) and last assessment completed at least one week after the first (n = 78 for depression and n = 47 for anxiety). Analyses were made separately for the Web-based sample and the university students. For the depression scores of the Web-based sample, there was a significant decrease over time (*P*< .0005) which was more marked for those who spent longer than a day between assessments (*P*< .0005). Similarly, anxiety scores decreased significantly (*P*< .0005), and to a large extent for those who had spent more than one day between assessments (*P*< .0005). Estimated marginal means are shown in Table 3. Due to the small numbers in the sample of university students (not shown), time spent between assessments was dichotomized to 0 or more days (the latter combining the categories of within a week, and over a week or more). For this sample, there was no significant change in depression (*P*= .852) or anxiety (*P*= .752) scores for those who spent more than a day between assessments compared to those who spent less than a day.

**Table 3:** Estimated marginal mean Goldberg Depression and Anxiety Scale scores (with 95% CI) for the MoodGYM web-based sample. Scores for the first and last assessments, by gender and by time between first and last assessments

	Score (95% CI)*			
	Goldberg Depression Scale		Goldberg Anxiety Scale	
	Males	Females	Males	Females
Assessments done same day				
First	4.88 (4.62-5.15)	5.35 (5.14-5.56)	5.26 (4.94-5.57)	5.77 (5.52-6.03)
Last	4.78 (4.50-5.05)	5.24 (5.02-5.46)	5.11 (4.77-5.44)	5.60 (5.33-5.88)
Assessments done within a week				
First	4.42 (3.03-5.80)	6.33 (5.20-7.46)	5.22 (3.53-6.91)	5.63 (3.84-7.41)
Last	3.08 (1.63-4.53)	5.06 (3.87-6.24)	4.56 (2.77-6.34)	5.25 (3.35-7.15)
Assessments done over a week or more				
First	4.67 (3.43-5.90)	6.27 (5.44-7.11)	5.80 (4.20-7.40)	6.63 (5.47-7.79)
Last	3.93 (2.64-5.23)	5.12 (4.25-6.00)	4.20 (2.50-5.90)	4.89 (3.66-6.13)
* CI indicates Confidence Interval				

## Discussion

Visitors who register on the MoodGYM Web site have high levels of anxiety and depression symptoms relative to population samples. For community registrants who choose to go through the training program, there is evidence that anxiety and depression symptoms resolve with progress across the modules. However, university students who start the intervention with low symptom levels show no change over the period. To evaluate the plausibility of the intervention and its "dose" effect, we examined change in scores between the first occasion of measurement and the last, independently of which modules were completed. Three periods were observed: less than one day between completing two assessments, last assessment within one week, and last assessment completed at least one week after the first. The findings from these analyses suggest that greater change in symptoms is associated with longer exposure to the site, as indexed by longer periods between completed assessments. However, given the small change that occurred over an interval of less than one day, the data are consistent with recent reports of the effectiveness of one-session cognitive behavior therapy interventions [18,19].

MoodGYM registrants decline on average 3 points over the 5 modules if all modules are completed. More specifically, Table 3 illustrates that users have average starting scores of between 6.33 and 4.42, and average post-intervention scores of between 3.08 and 5.24. The significance of these changes can be determined by both examining the distribution of anxiety and depression scores in appropriate population samples [13] and the highest scores of individuals who are likely to be clinical cases. Given the prevalence of clinical depression is about 7% in Australia [20], those scoring at a level to reach the top 10% range might be regarded as meeting or nearly meeting clinical criteria. For young people (aged 20-24 years) a drop from a score of 6 to 3, indicates a shift from a percentile rank of 79.4 to that of 38.1. For a person aged 40-44, the drop corresponds to a drop of 90.2 to a rank of 63.8. These data suggest substantial shifts down from high (but not clinical) levels for the younger users, and shifts from clinical levels in older adults.

Due to the limitations of the present design, we cannot conclude that the training program was responsible for the changes in mental health symptoms. Randomized controlled trials are necessary to evaluate MoodGYM and other psychological interventions on the Internet relative to both waitlist control conditions and standard treatments. Because such methodology was not employed, it is difficult to know whether the changes were due to depressive symptoms resolving over time [21]. Regression to the mean may also explain the findings. Selection (or self-selection) on the basis of high symptoms at a particular time will result in reversion to more normal levels on a second testing. Moreover, individuals with fewer mental health problems may be differentially inclined to fill in questionnaires in later modules in the site. Nevertheless, the findings from the study demonstrate the feasibility and highlight the potential public health implications of Internet use in mental health. From a public health perspective, the use of the Web in treatment, prevention, and promotion is likely to increase enormously given its potential for providing services for those who do not seek or cannot obtain help from health professionals for reasons of cost, lack of accessibility, or the perceived stigma associated with seeking professional help.

The use of community-collected Web data raises interesting methodological, epidemiological, and statistical issues. It is difficult to identify the population to which samples refer when there is no clear sampling frame or method of sampling and where there is no direct subject contact. Appropriate methods to deal with the vast amount of incomplete and missing data are needed. If we can assume data are missing at random (MAR) [22] if not missing completely at random (MCAR), we need to collect data to describe the incomplete and missing data that can be incorporated in appropriate methods of analysis (eg, Full Information Maximum Likelihood Methods) [22]. Finally, the suitability of intention to treat analyses in the context of large-scale community Web interventions (where adherence to the training program may be neither desirable nor achievable), requires careful consideration.

To date, mental health Web sites have been found to be useful for screening the public for depression using the Centers for Epidemiological Studies Depression (CES-D) scale [9]. There is some evidence that Web sites may be a useful adjunct to treatment in clinical settings [10,23]. However, to our knowledge there has been no previous published evidence concerning the impact of a Web-based therapy intervention on the mental health of community users.

MoodGYM illustrates the means by which the Internet might be harnessed to prevent depression, and early results from the site point to the public health potential of mental health Web sites. At the time of writing, MoodGYM was ranked 15th of about 1790 sites in Google's "Mood" subcategory, indicating that it is popular and linked to other "high quality sites" [24]. It may be of practical interest to general practitioners in all countries since it provides a free service that might, like cognitive behavioral bibliotherapy, be used as an adjunct to standard consultation.

## Abbreviations

CBT: Cognitive Behavior Therapy
CI: Confidence Interval
SD: Standard Deviation

## Multimedia Appendix

Downloadable presentation with screenshots of "MoodGYM"
