# Supplementary material for: Web-based Cognitive Behavior Therapy: Analysis of Site Usage and Changes in Depression and Anxiety Scores
Source: J Med Internet Res. 2002 Feb 15;4(1):e3. doi: 10.2196/jmir.4.1.e3 (PMC1761927; doi:10.2196/jmir.4.1.e3)
Supplement: Supplementary file 1 [file jmir_v4i1e3_app1.ppt]

## Slide 1
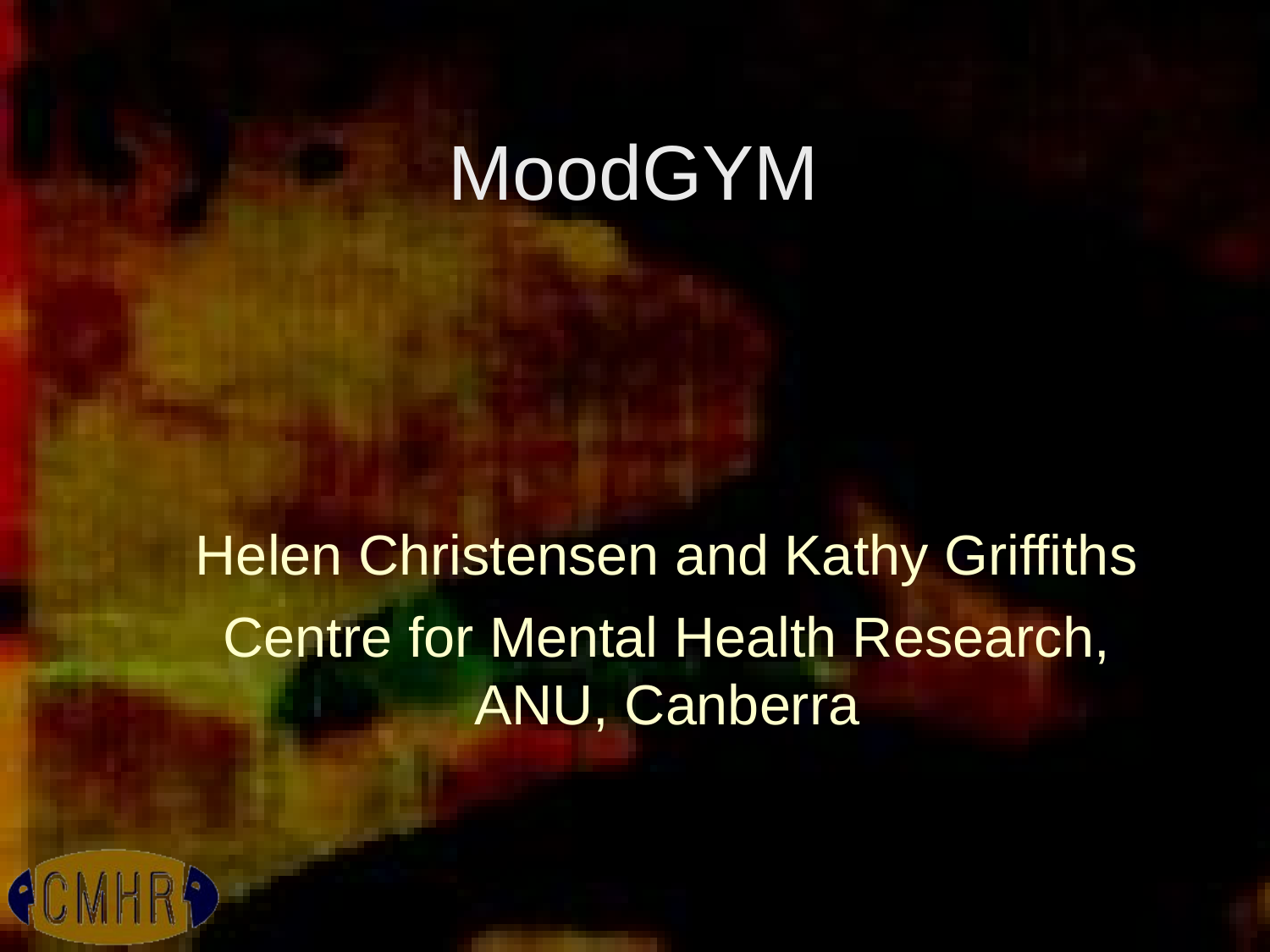

# MoodGYM
Helen Christensen and Kathy Griffiths
Centre for Mental Health Research, ANU, Canberra

## Slide 2
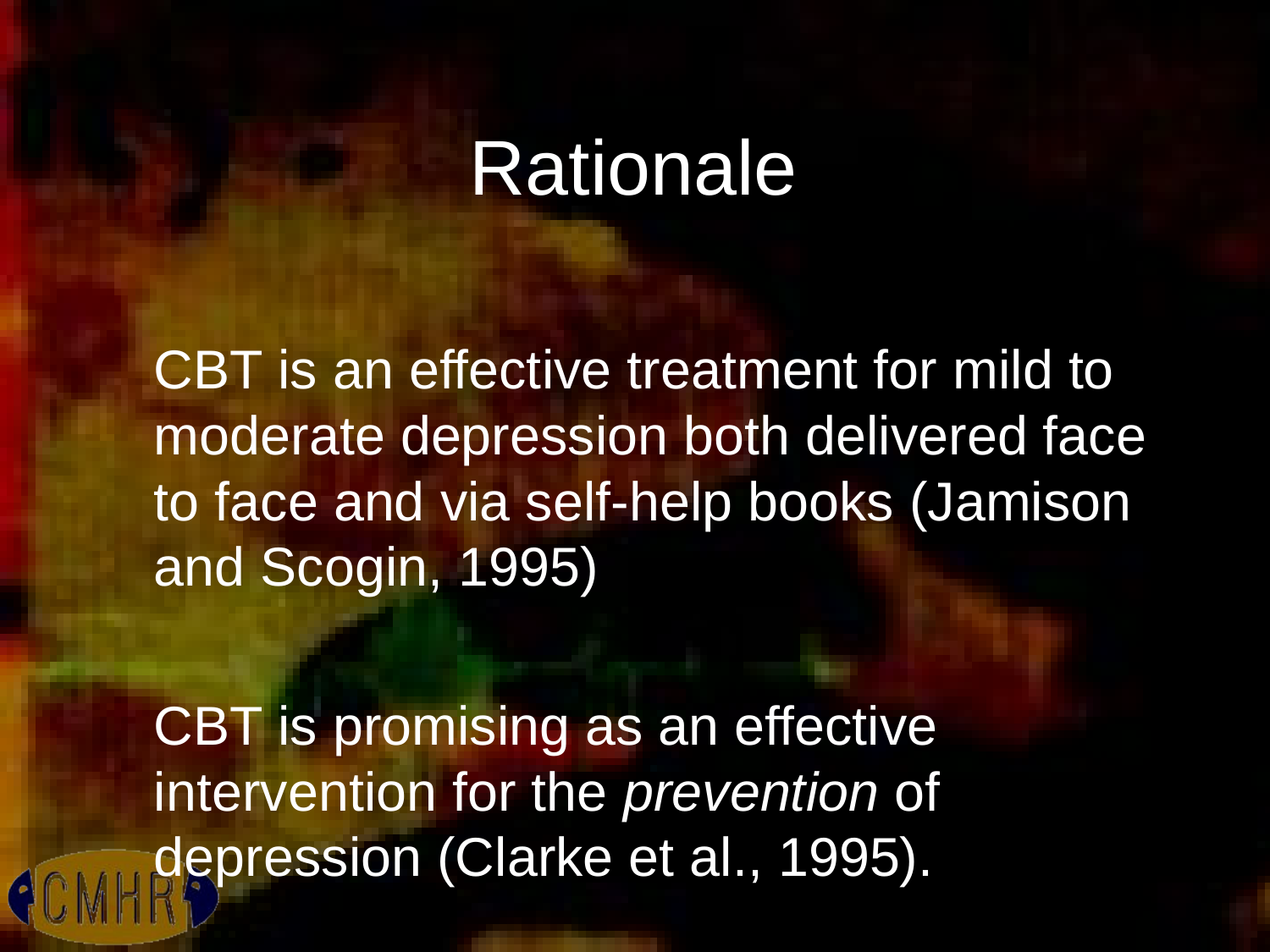

# Rationale
CBT is an effective treatment for mild to moderate depression both delivered face to face and via self-help books (Jamison and Scogin, 1995)
CBT is promising as an effective intervention for the prevention of depression (Clarke et al., 1995).

## Slide 3
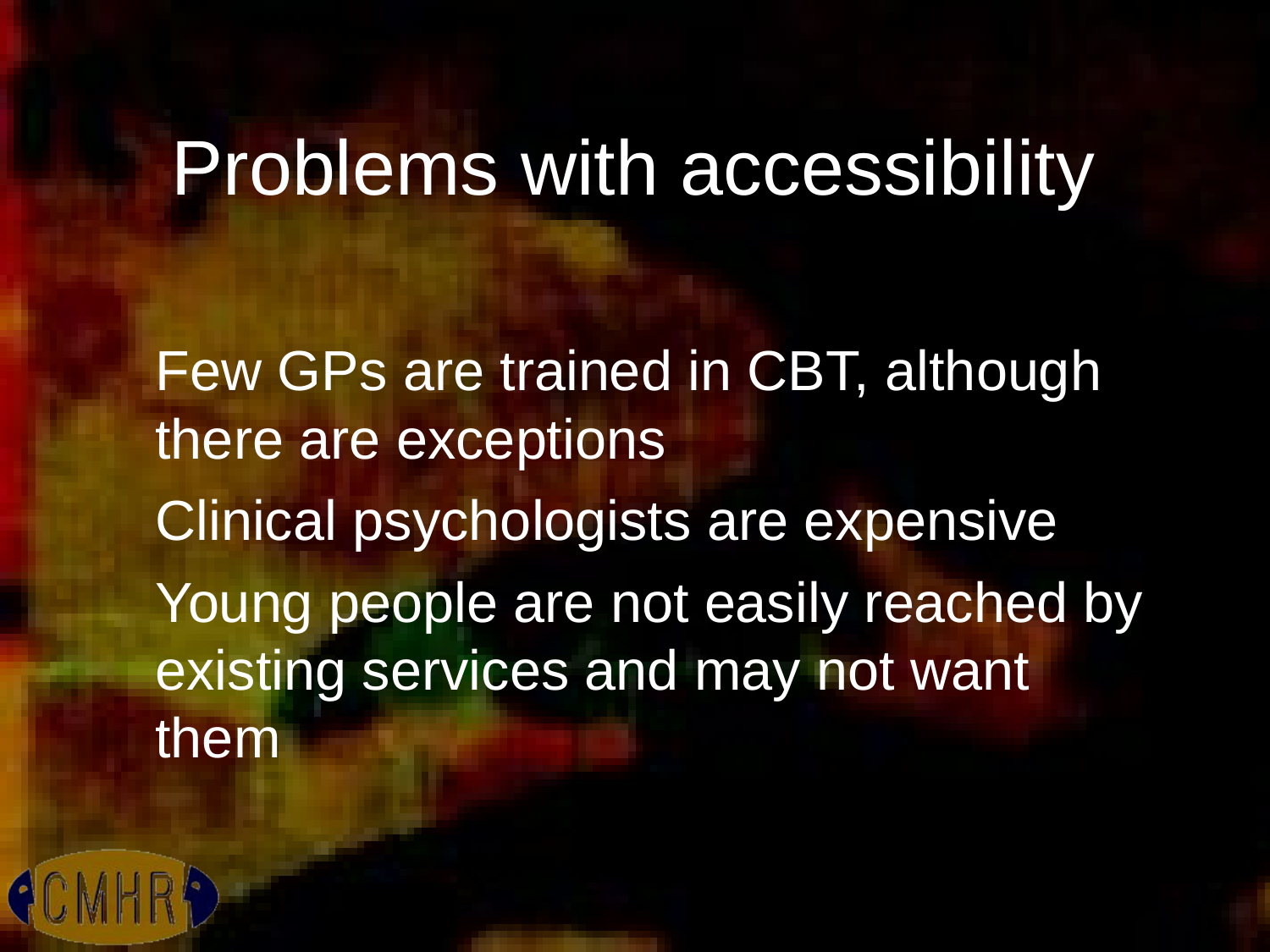

# Problems with accessibility
Few GPs are trained in CBT, although there are exceptions
Clinical psychologists are expensive
Young people are not easily reached by existing services and may not want them

## Slide 4
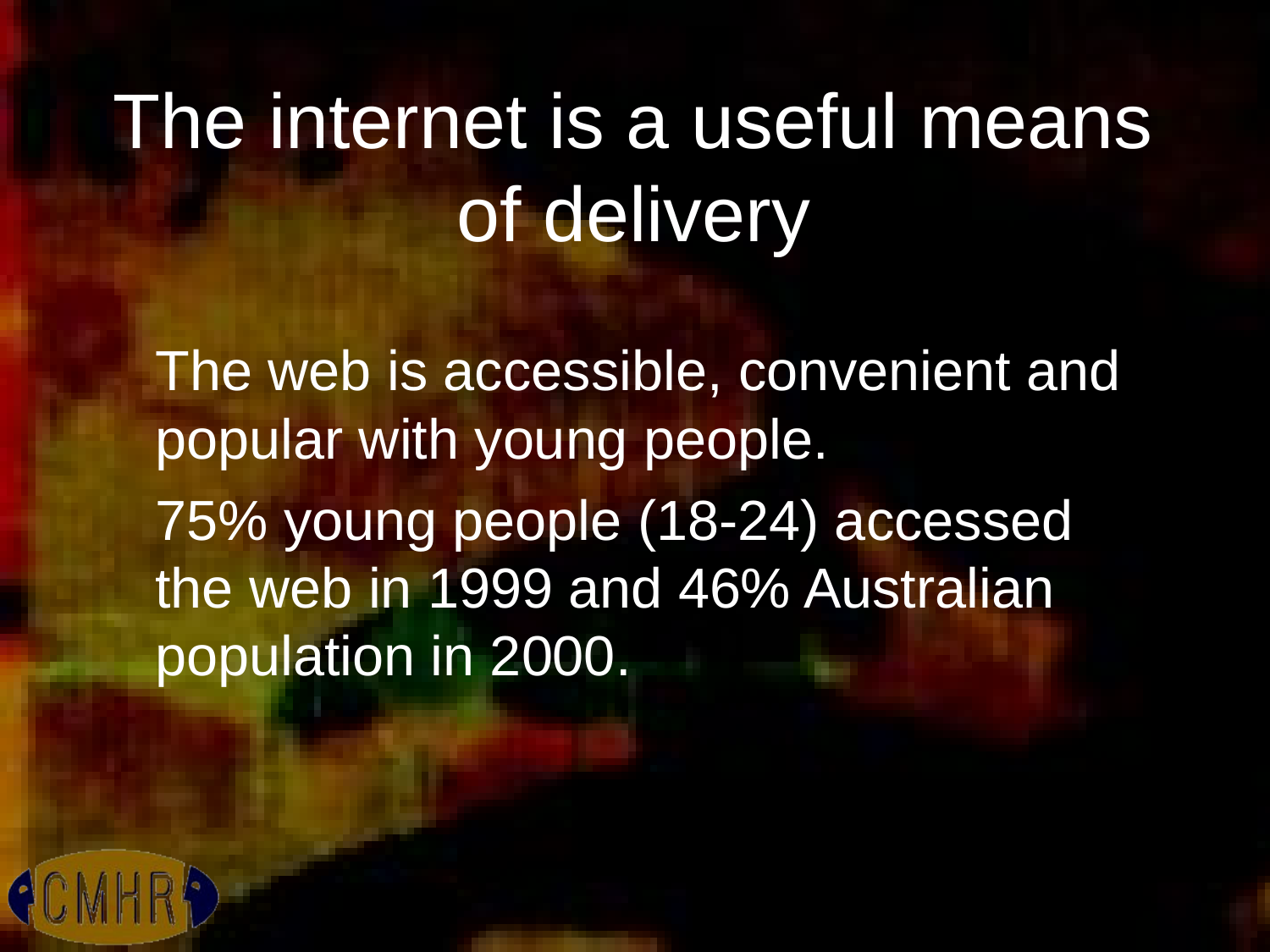

# The internet is a useful means of delivery
The web is accessible, convenient and popular with young people.
75% young people (18-24) accessed the web in 1999 and 46% Australian population in 2000.

## Slide 5
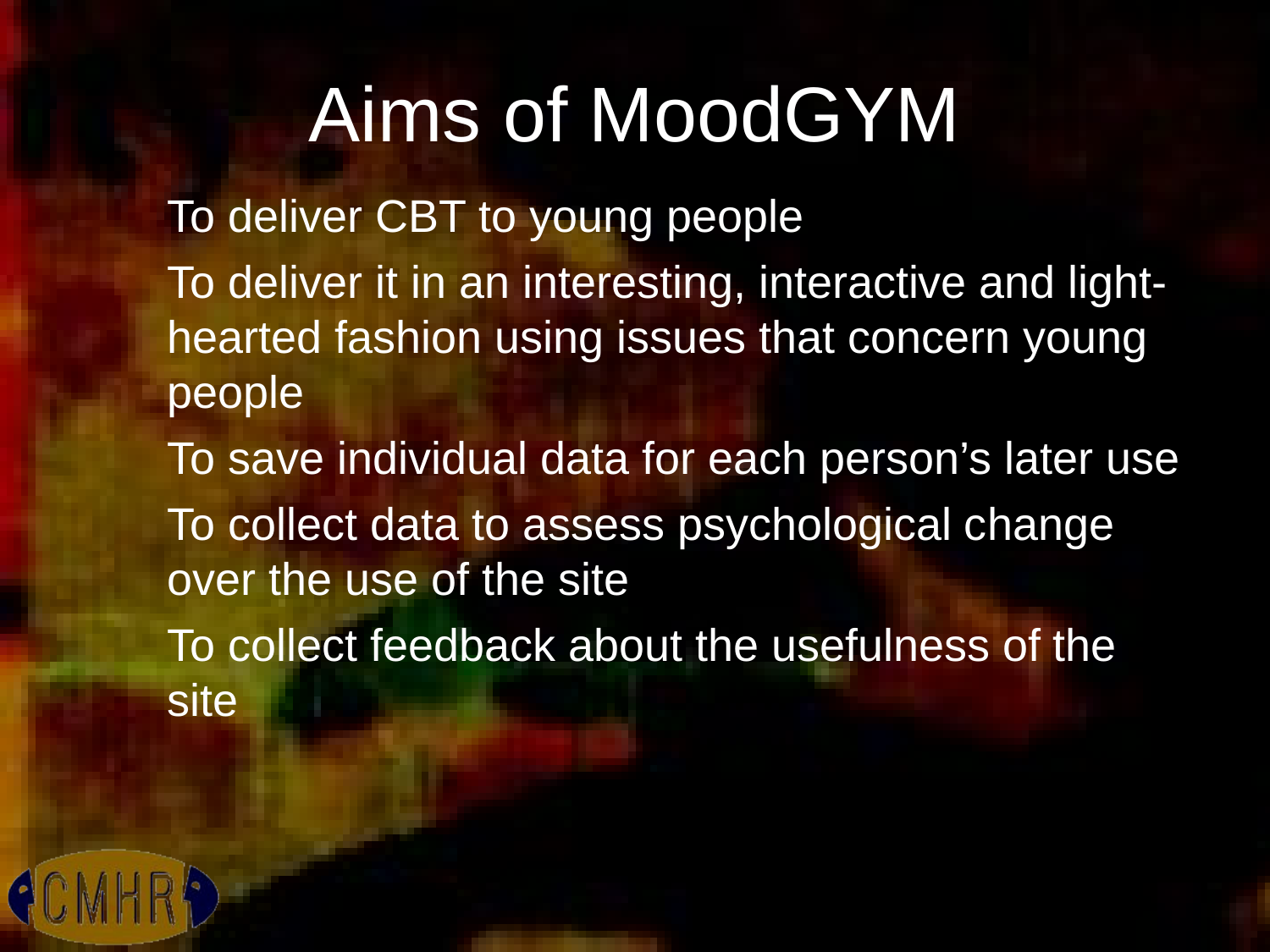

# Aims of MoodGYM
To deliver CBT to young people
To deliver it in an interesting, interactive and light-hearted fashion using issues that concern young people
To save individual data for each person’s later use
To collect data to assess psychological change over the use of the site
To collect feedback about the usefulness of the site

## Slide 6
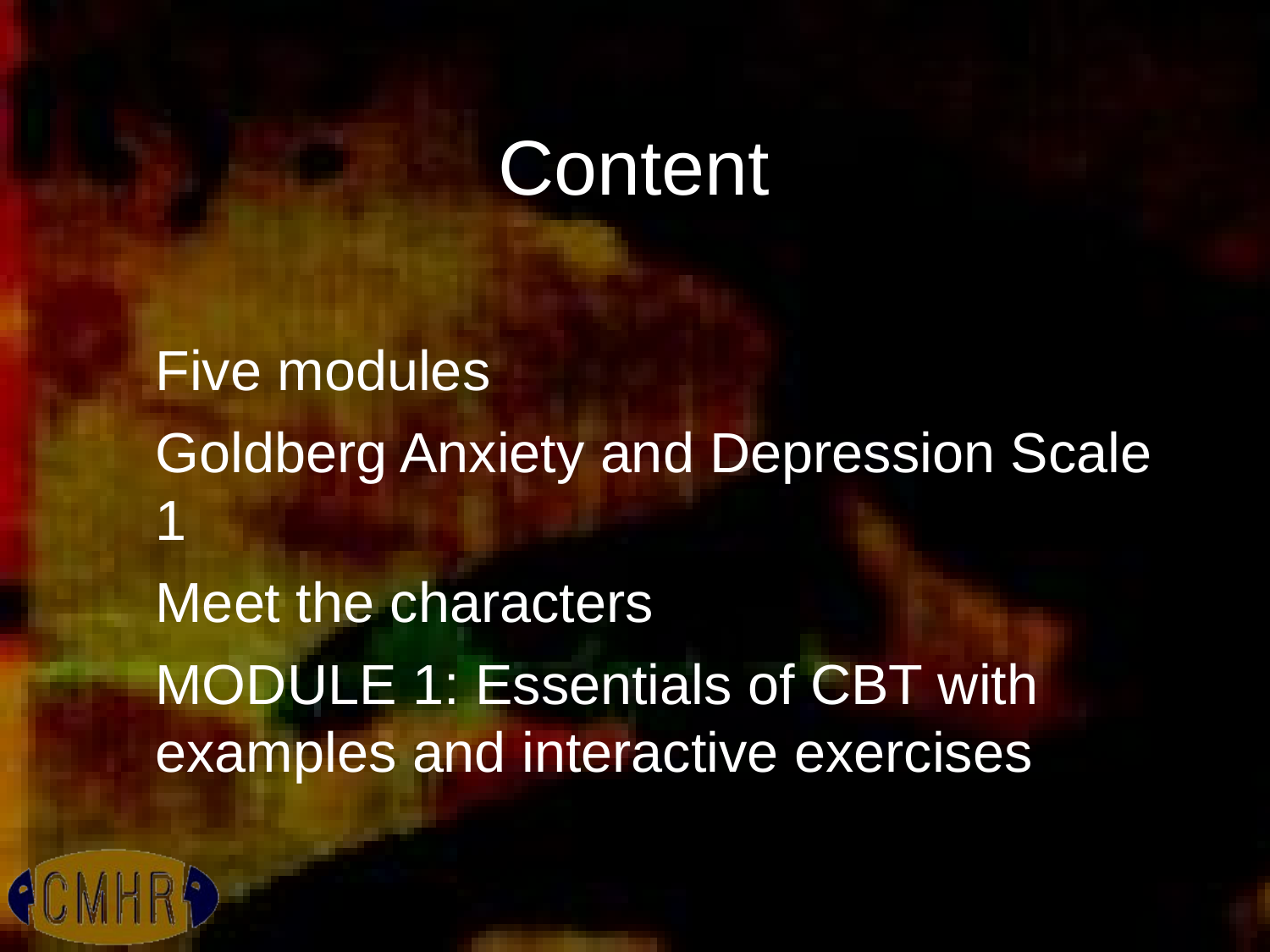

# Content
Five modules
Goldberg Anxiety and Depression Scale 1
Meet the characters
MODULE 1: Essentials of CBT with examples and interactive exercises

## Slide 7
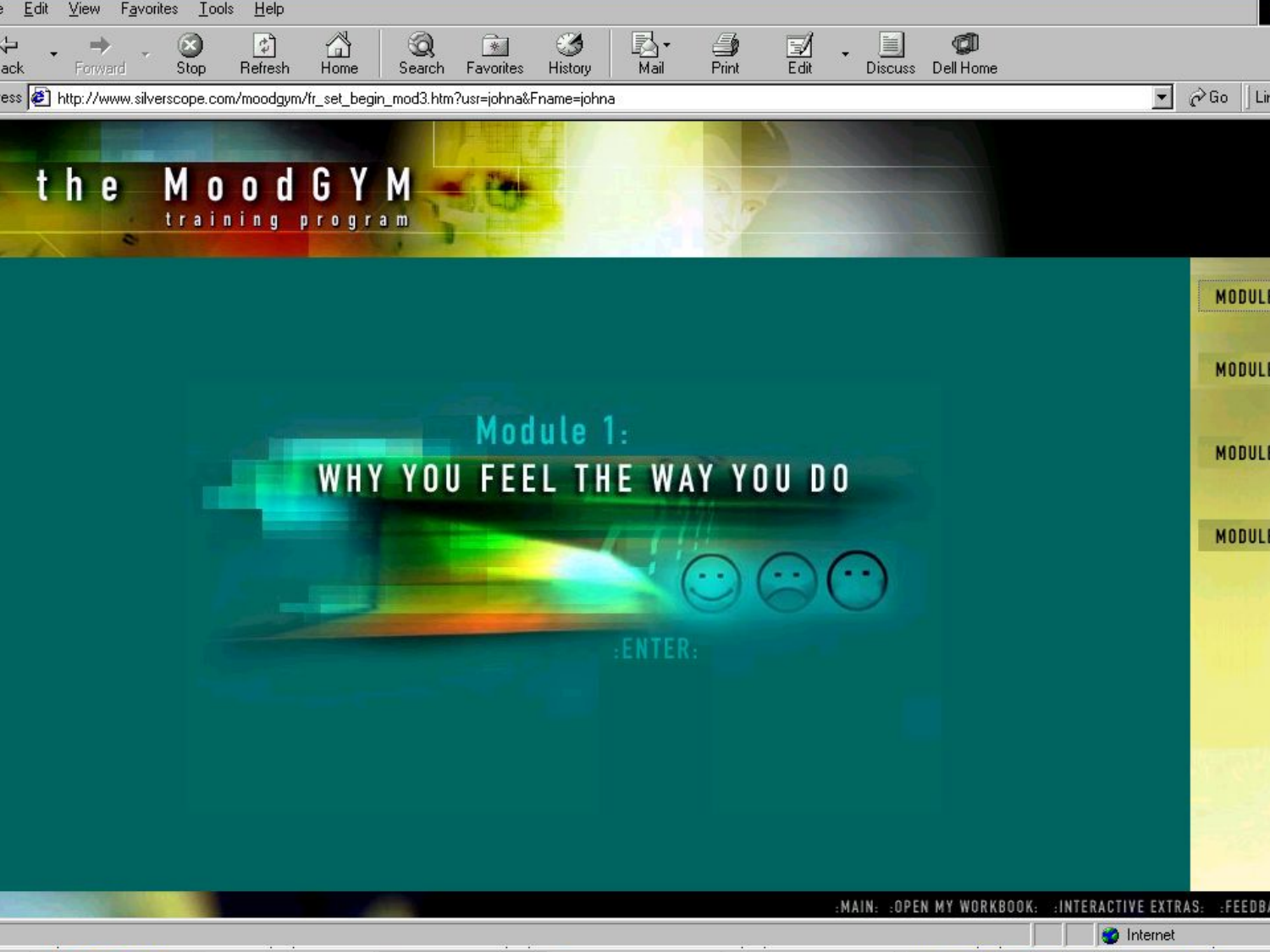

## Slide 8
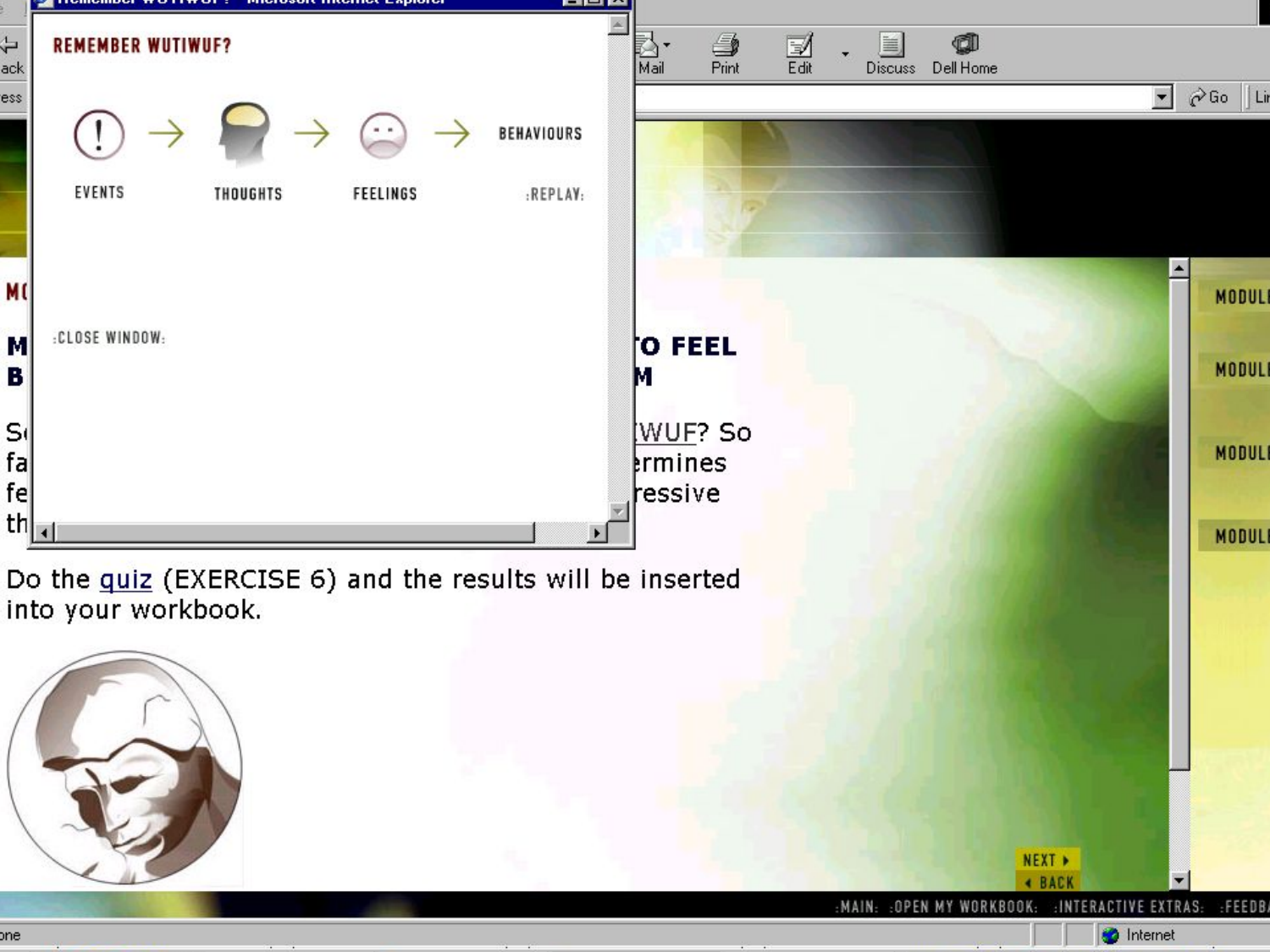

## Slide 9
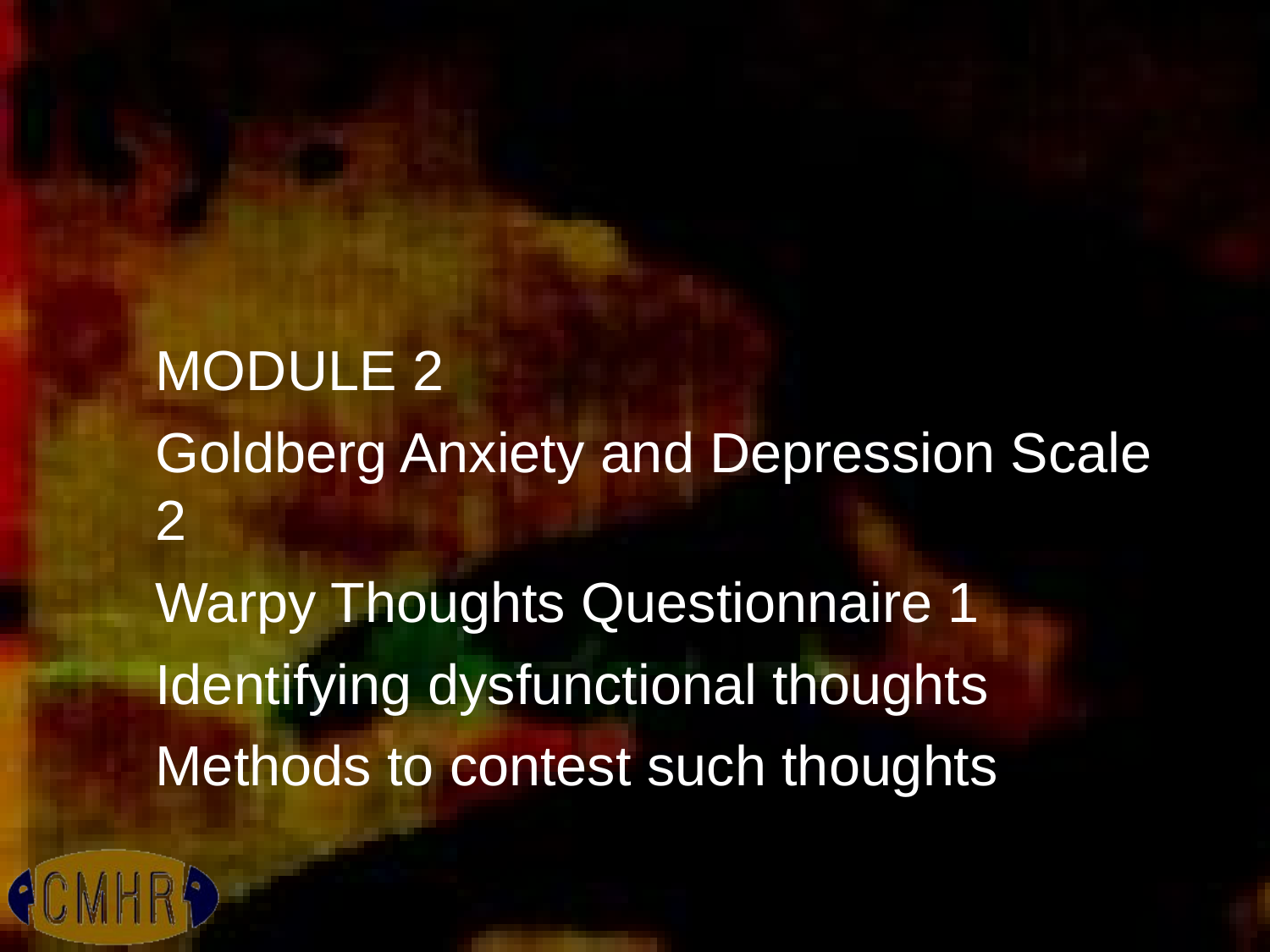

#
MODULE 2
Goldberg Anxiety and Depression Scale 2
Warpy Thoughts Questionnaire 1
Identifying dysfunctional thoughts
Methods to contest such thoughts

## Slide 10
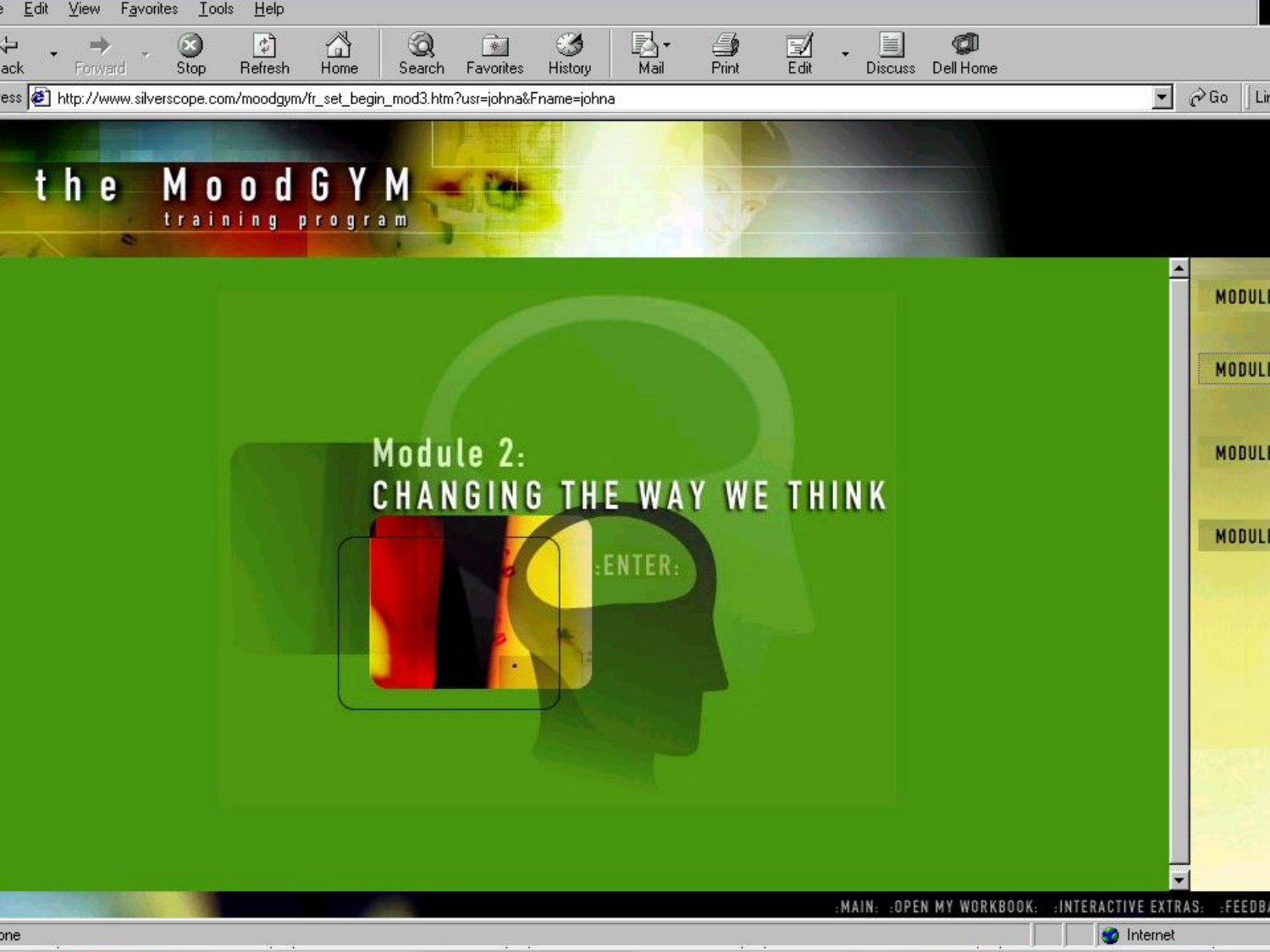

## Slide 11
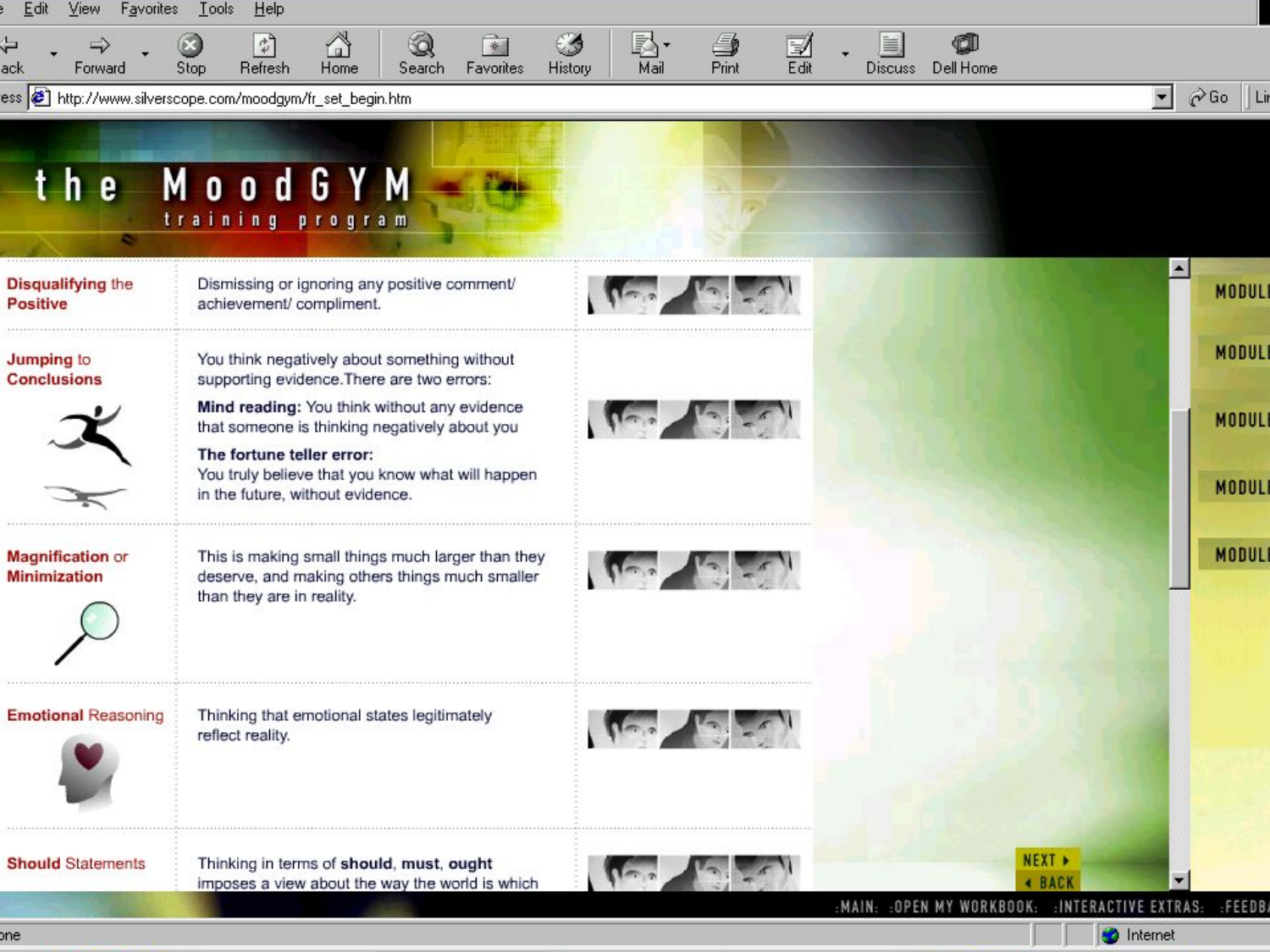

## Slide 12
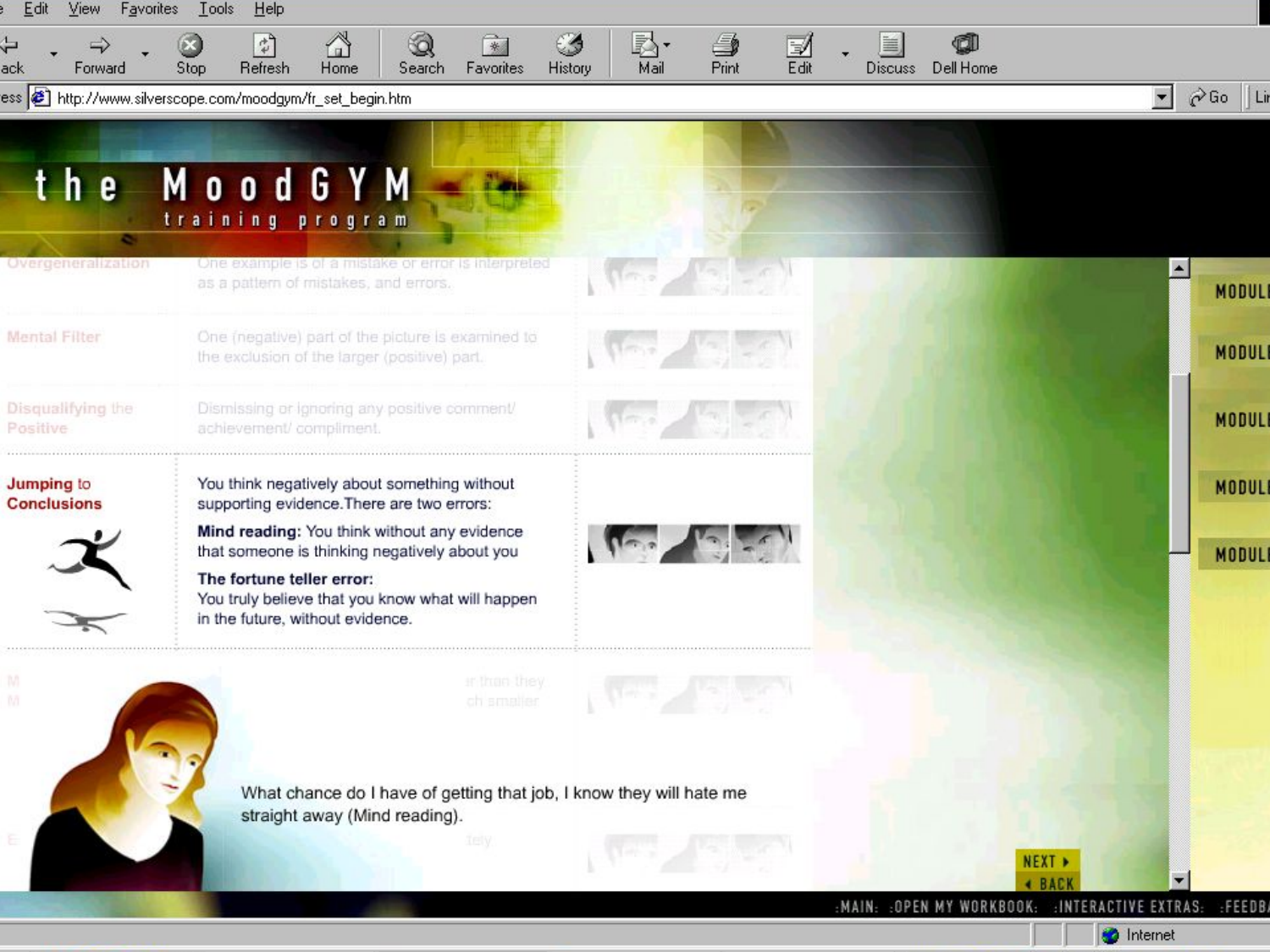

## Slide 13
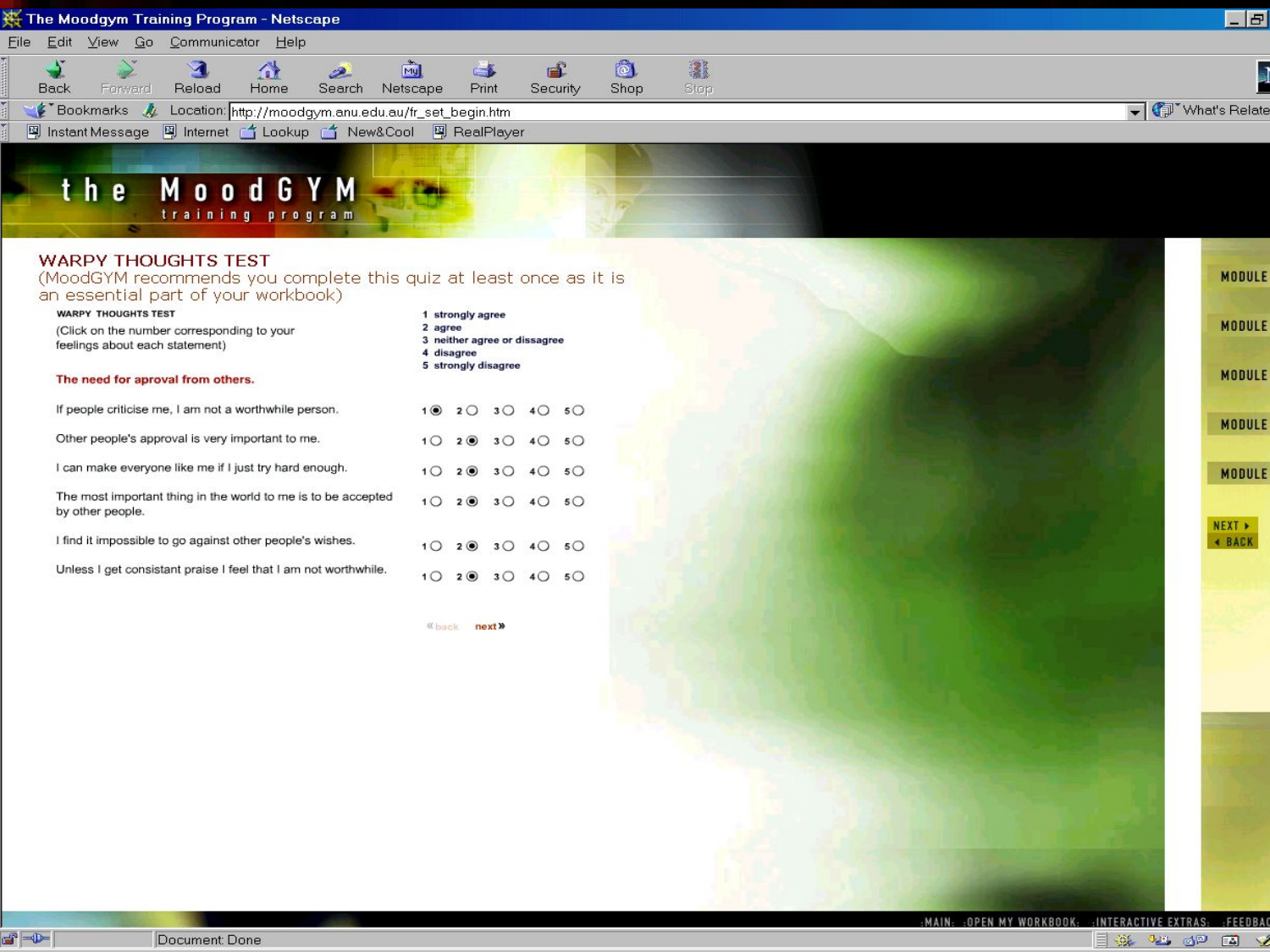

## Slide 14
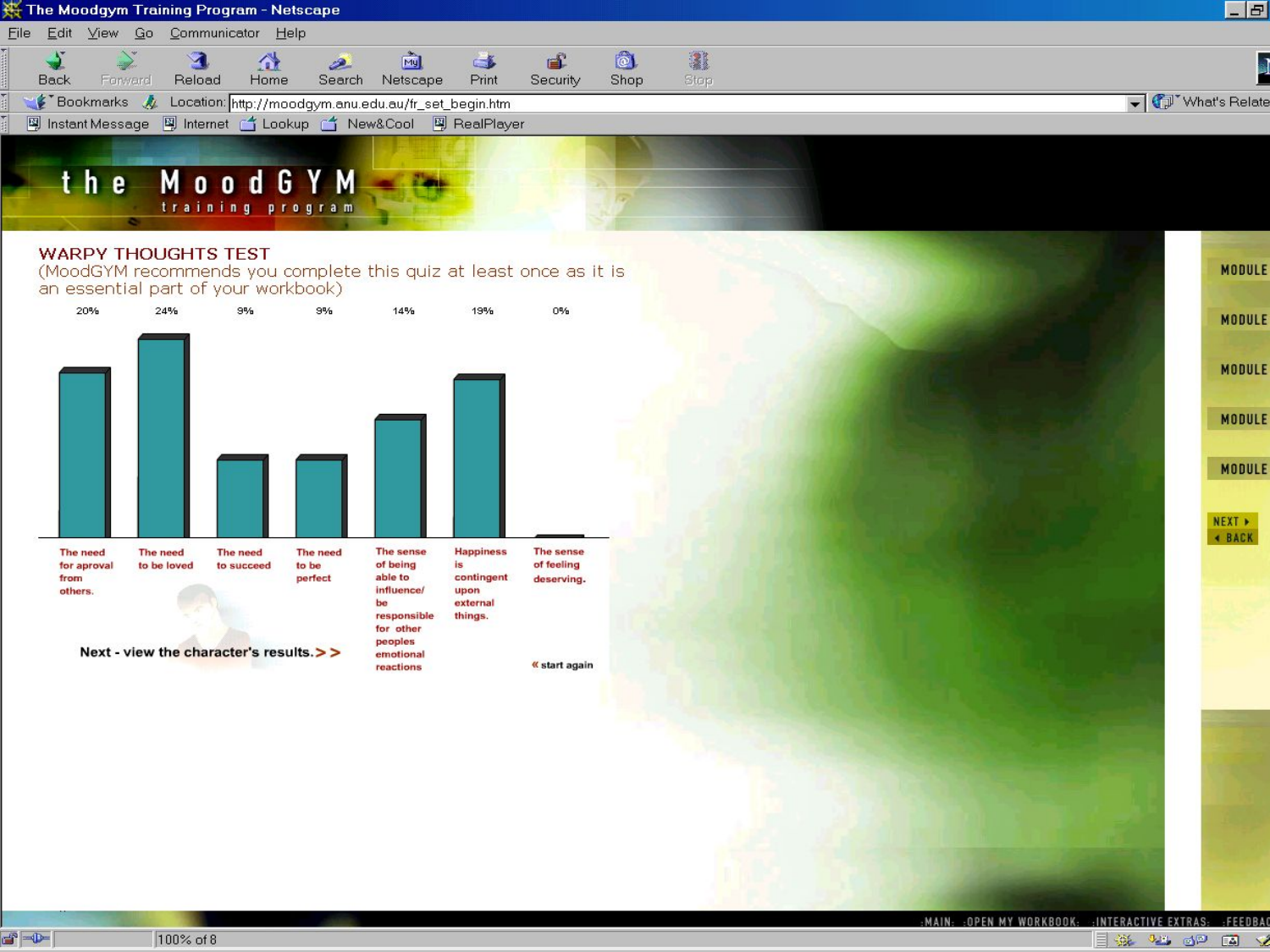

## Slide 15
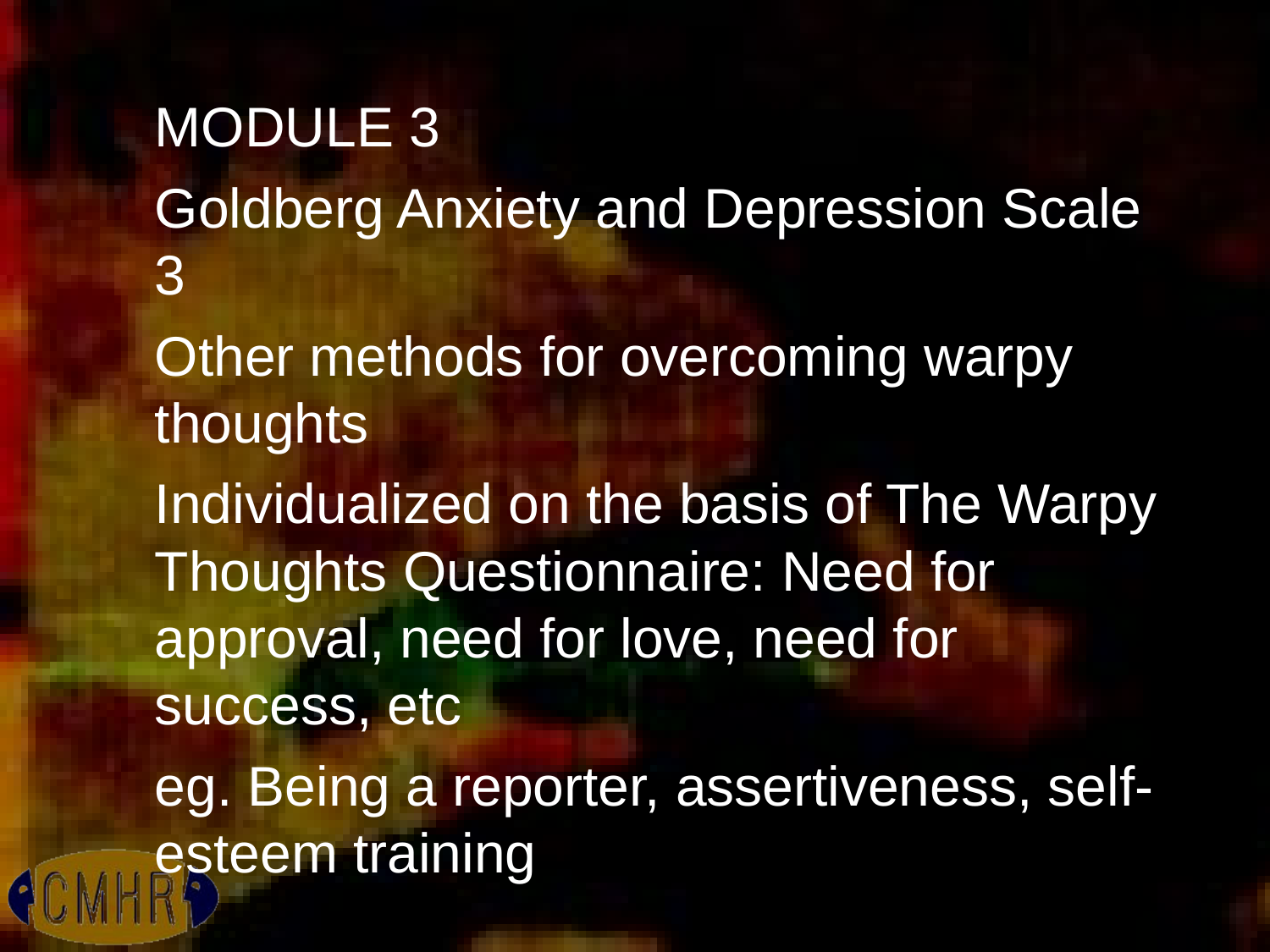

#
MODULE 3
Goldberg Anxiety and Depression Scale 3
Other methods for overcoming warpy thoughts
Individualized on the basis of The Warpy Thoughts Questionnaire: Need for approval, need for love, need for success, etc
eg. Being a reporter, assertiveness, self-esteem training

## Slide 16
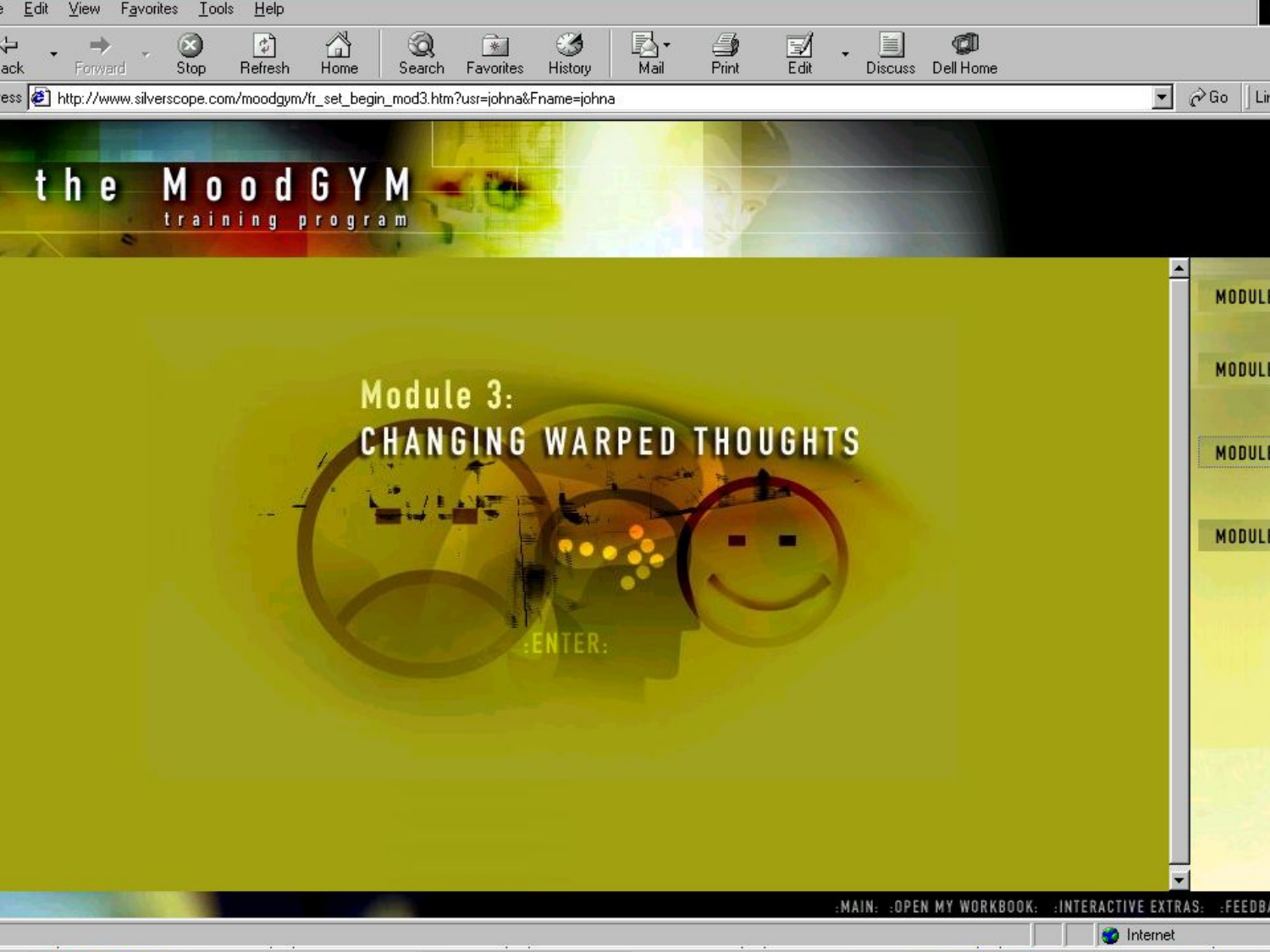

## Slide 17
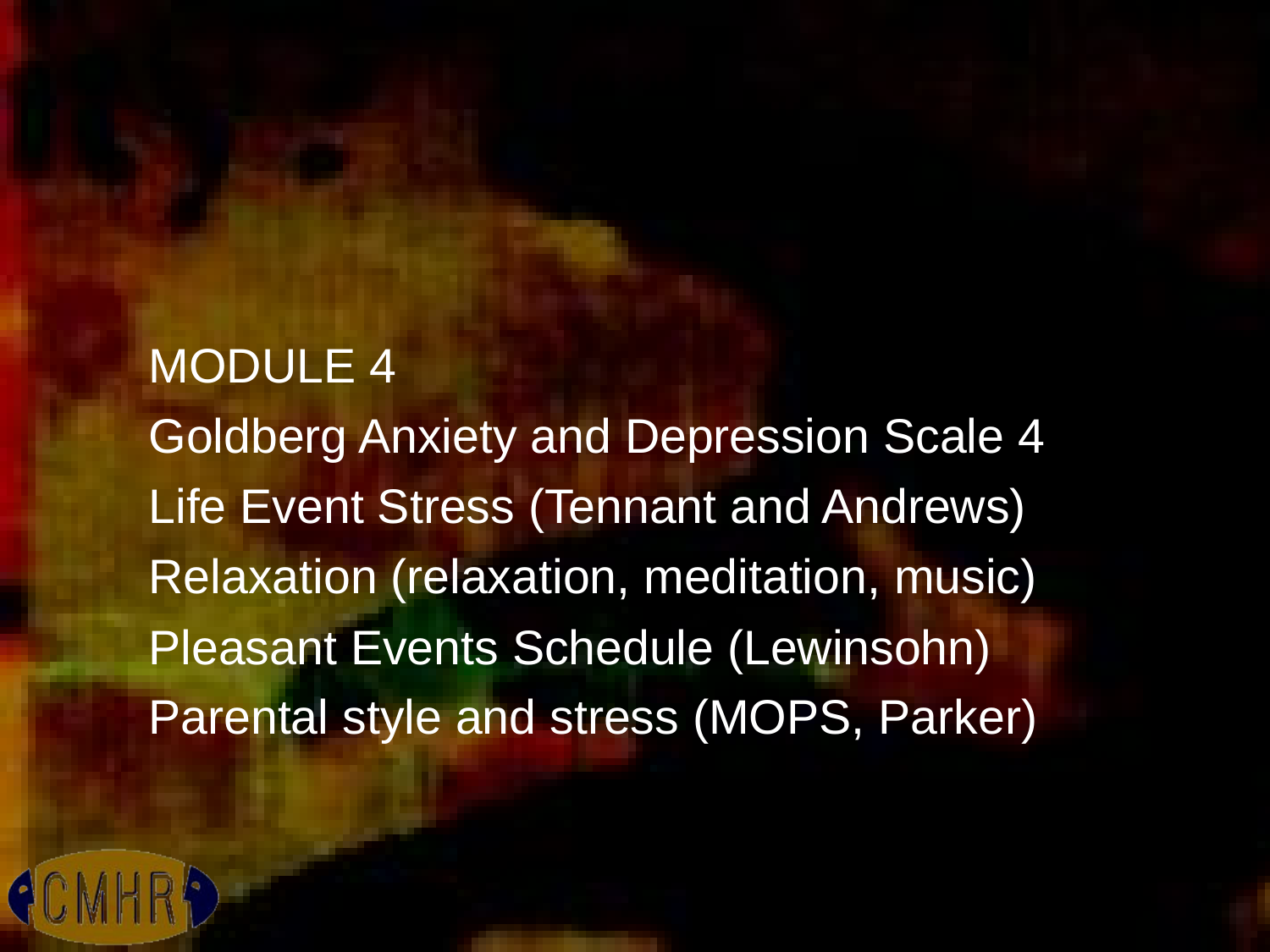

#
MODULE 4
Goldberg Anxiety and Depression Scale 4
Life Event Stress (Tennant and Andrews)
Relaxation (relaxation, meditation, music)
Pleasant Events Schedule (Lewinsohn)
Parental style and stress (MOPS, Parker)

## Slide 18
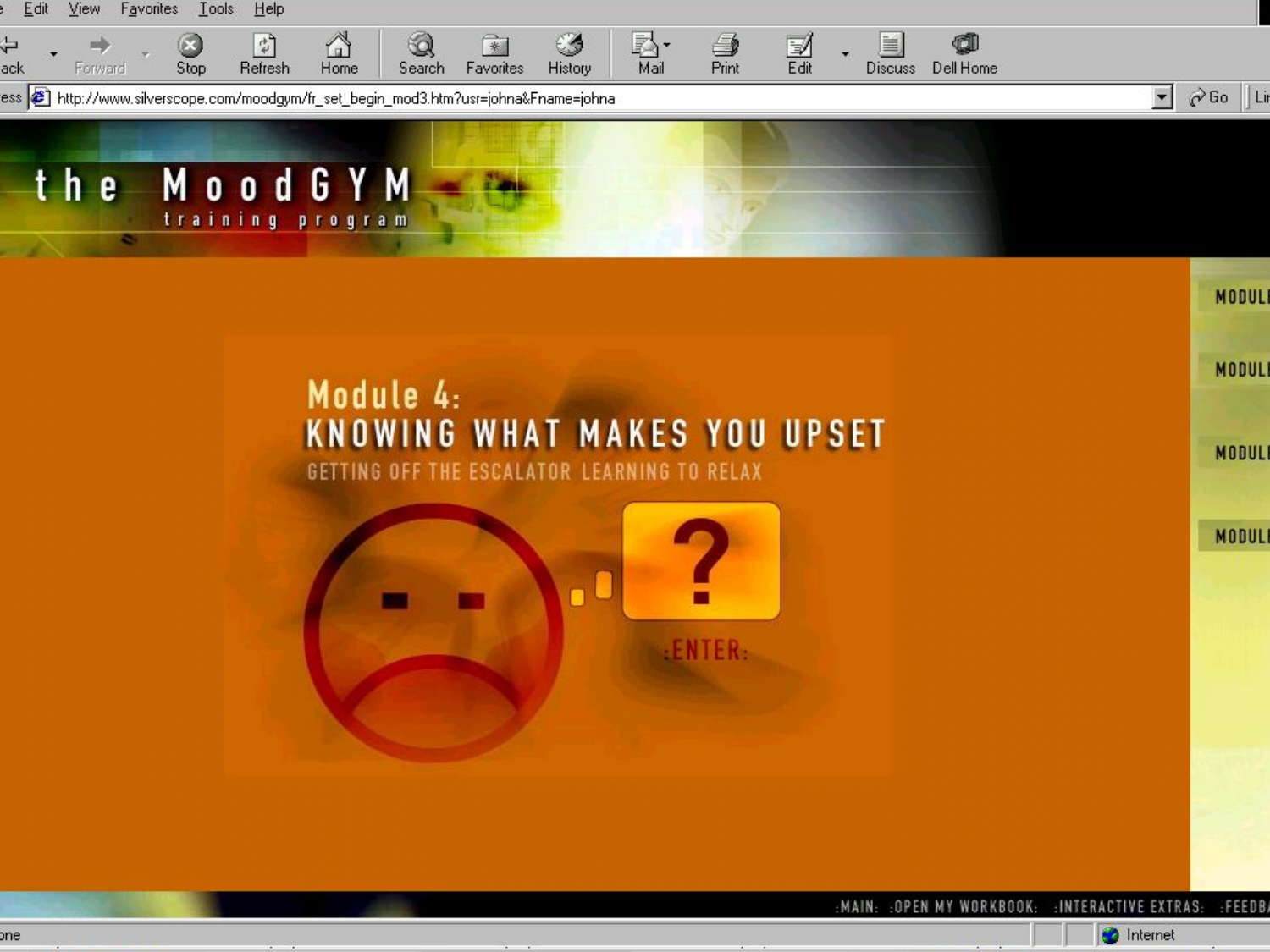

## Slide 19
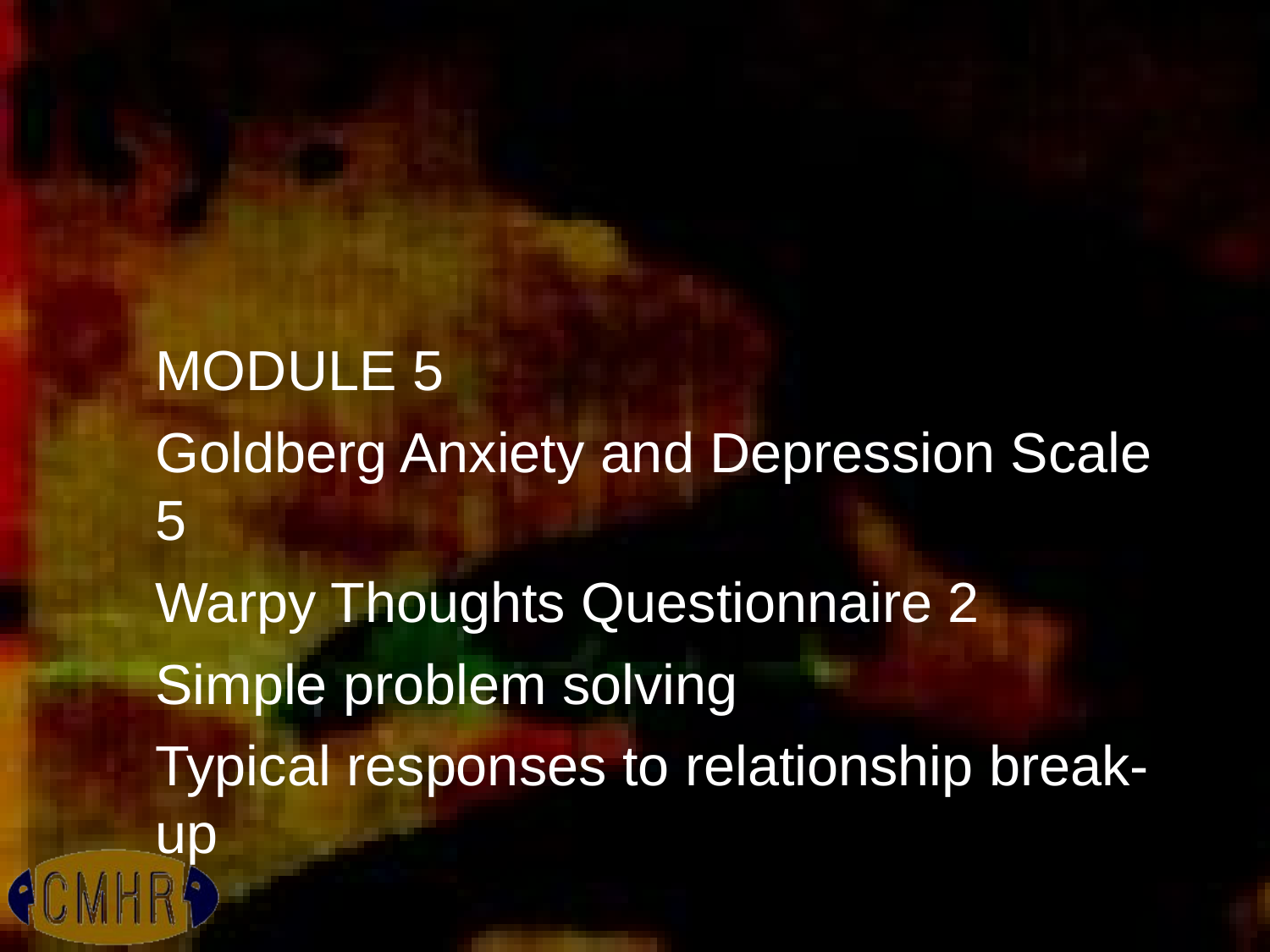

#
MODULE 5
Goldberg Anxiety and Depression Scale 5
Warpy Thoughts Questionnaire 2
Simple problem solving
Typical responses to relationship break-up

## Slide 20
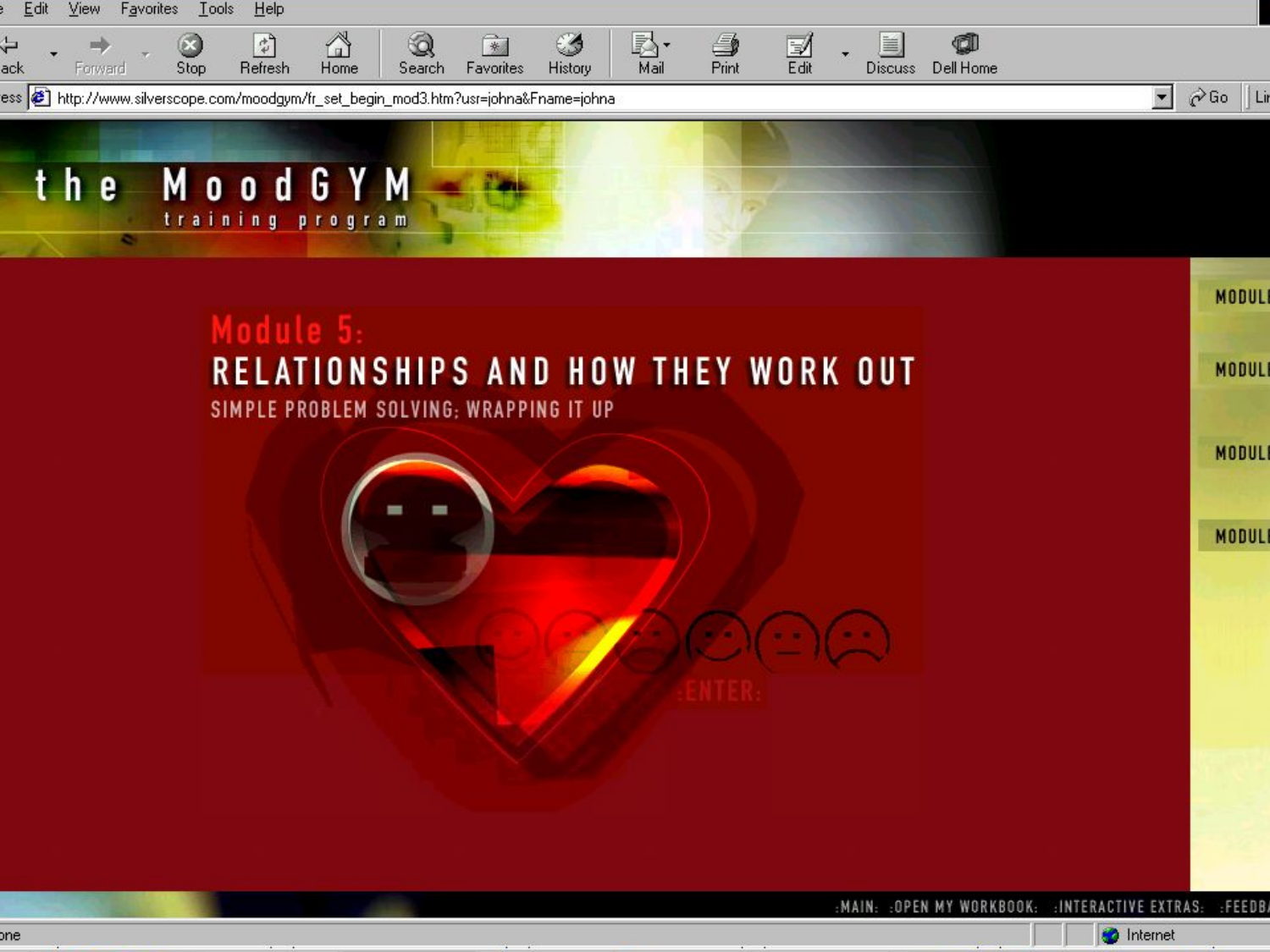

## Slide 21
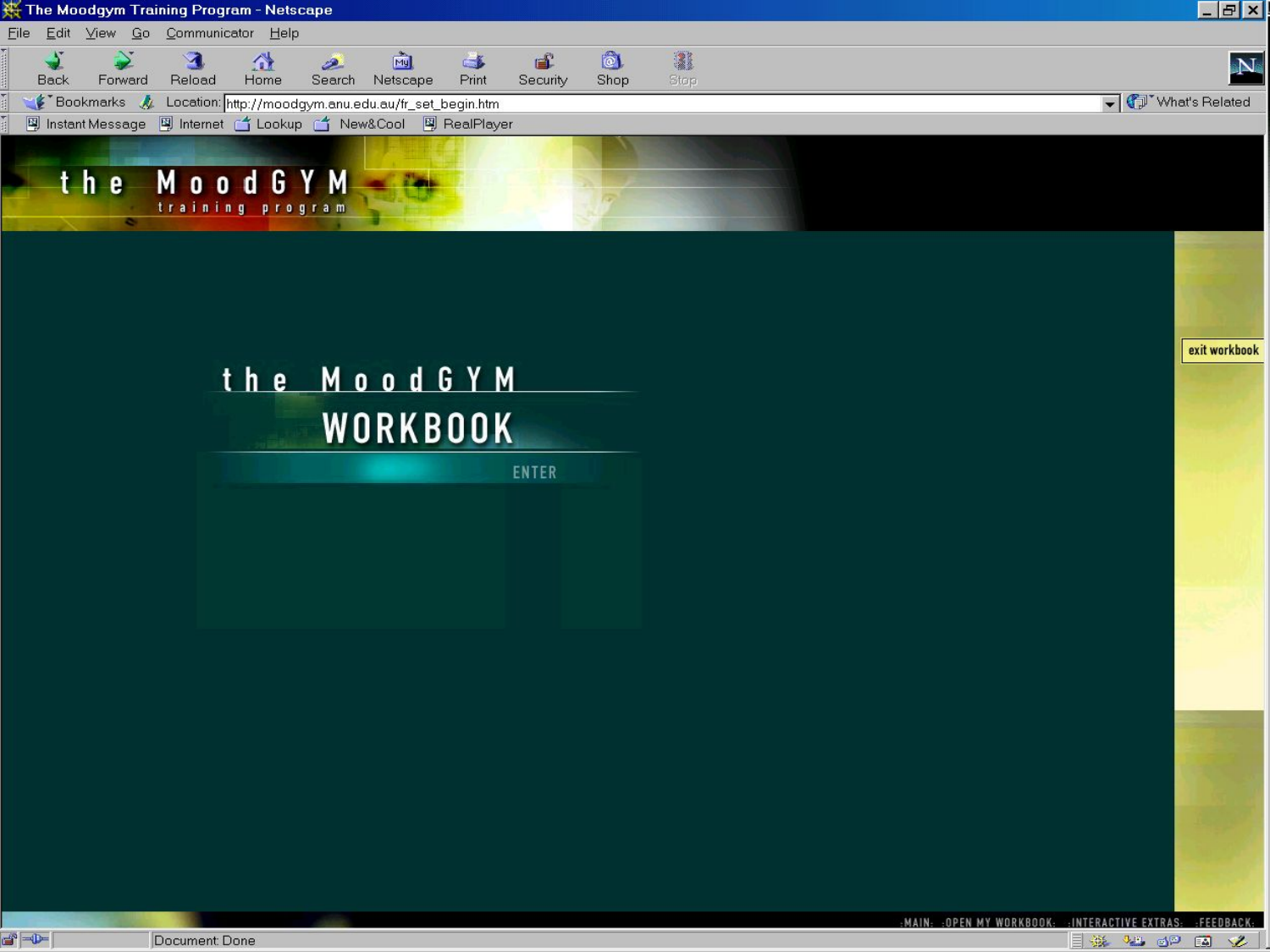

## Slide 22
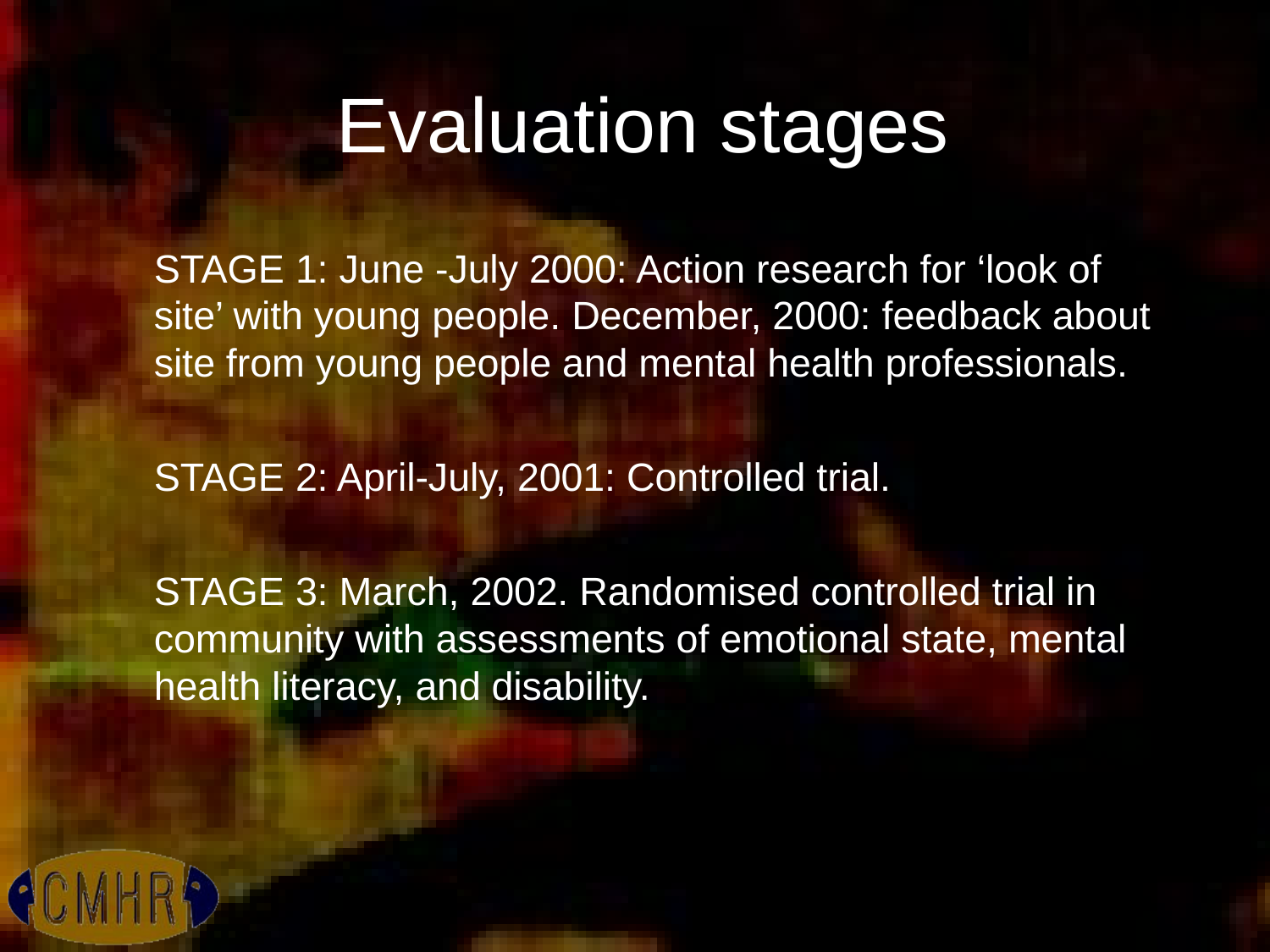

# Evaluation stages
STAGE 1: June -July 2000: Action research for ‘look of site’ with young people. December, 2000: feedback about site from young people and mental health professionals.
STAGE 2: April-July, 2001: Controlled trial.
STAGE 3: March, 2002. Randomised controlled trial in community with assessments of emotional state, mental health literacy, and disability.

## Slide 23
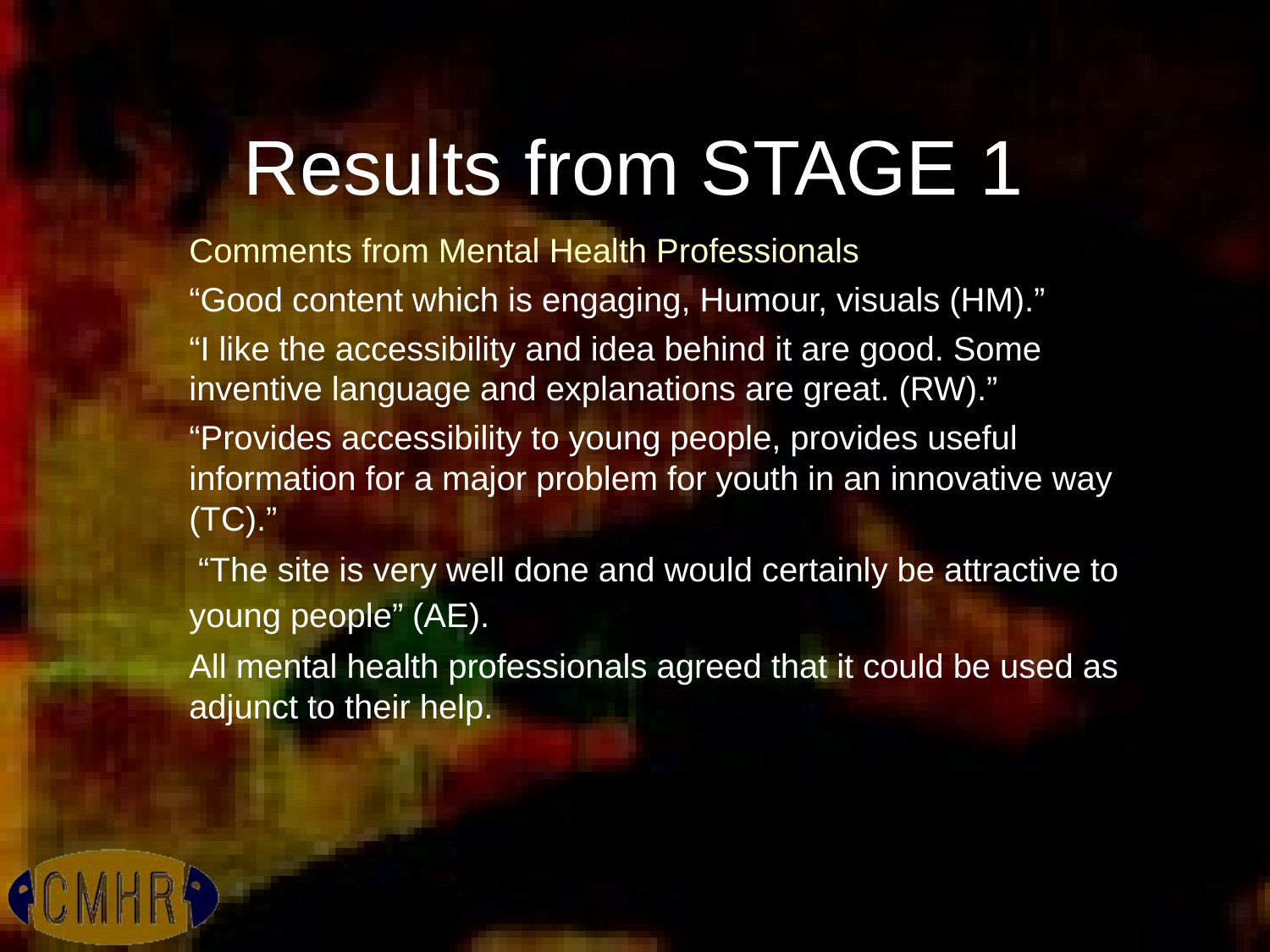

# Results from STAGE 1
Comments from Mental Health Professionals
“Good content which is engaging, Humour, visuals (HM).”
“I like the accessibility and idea behind it are good. Some inventive language and explanations are great. (RW).”
“Provides accessibility to young people, provides useful information for a major problem for youth in an innovative way (TC).”
 “The site is very well done and would certainly be attractive to young people” (AE).
All mental health professionals agreed that it could be used as adjunct to their help.

## Slide 24
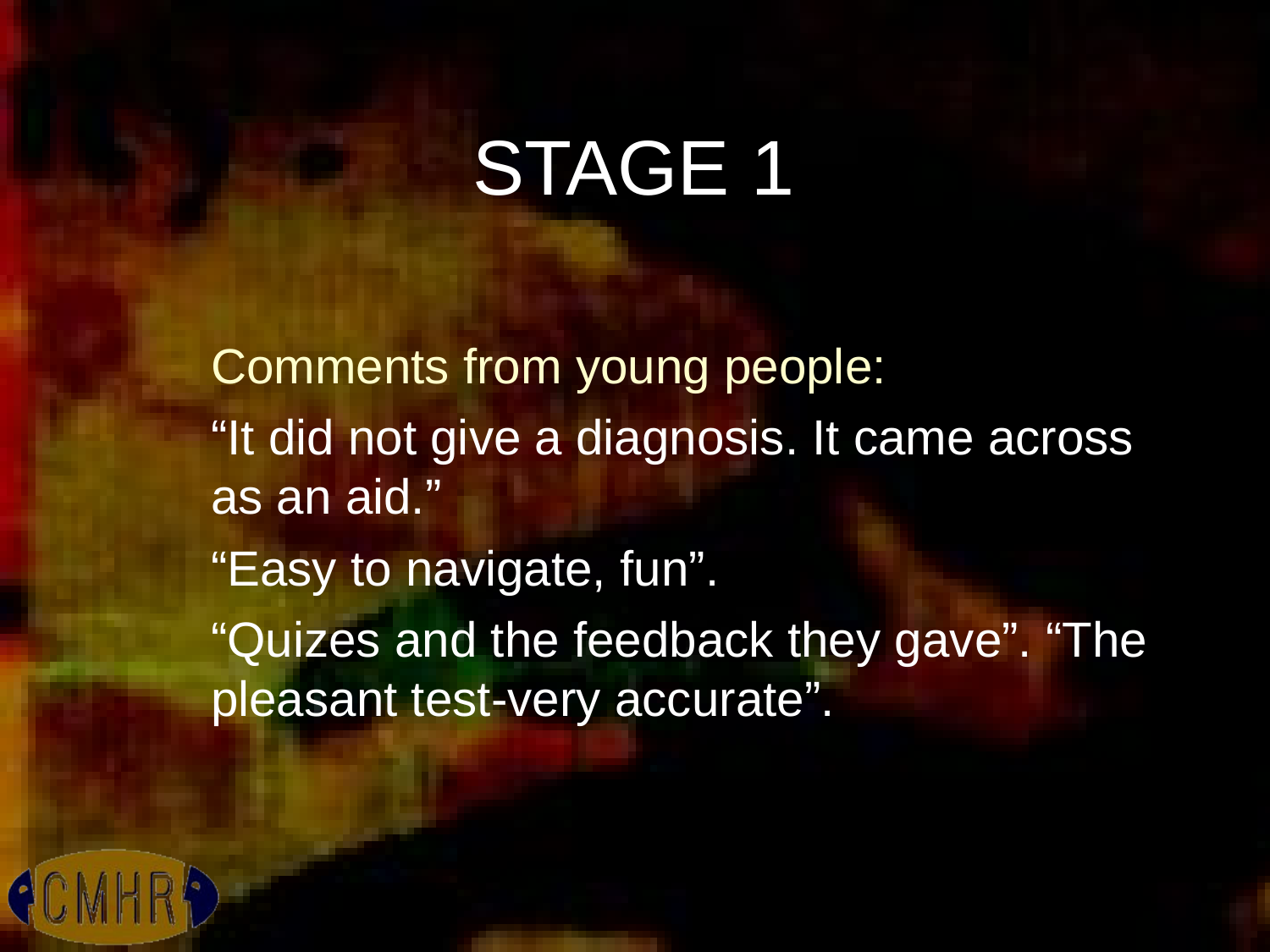

# STAGE 1
Comments from young people:
“It did not give a diagnosis. It came across as an aid.”
“Easy to navigate, fun”.
“Quizes and the feedback they gave”. “The pleasant test-very accurate”.

## Slide 25
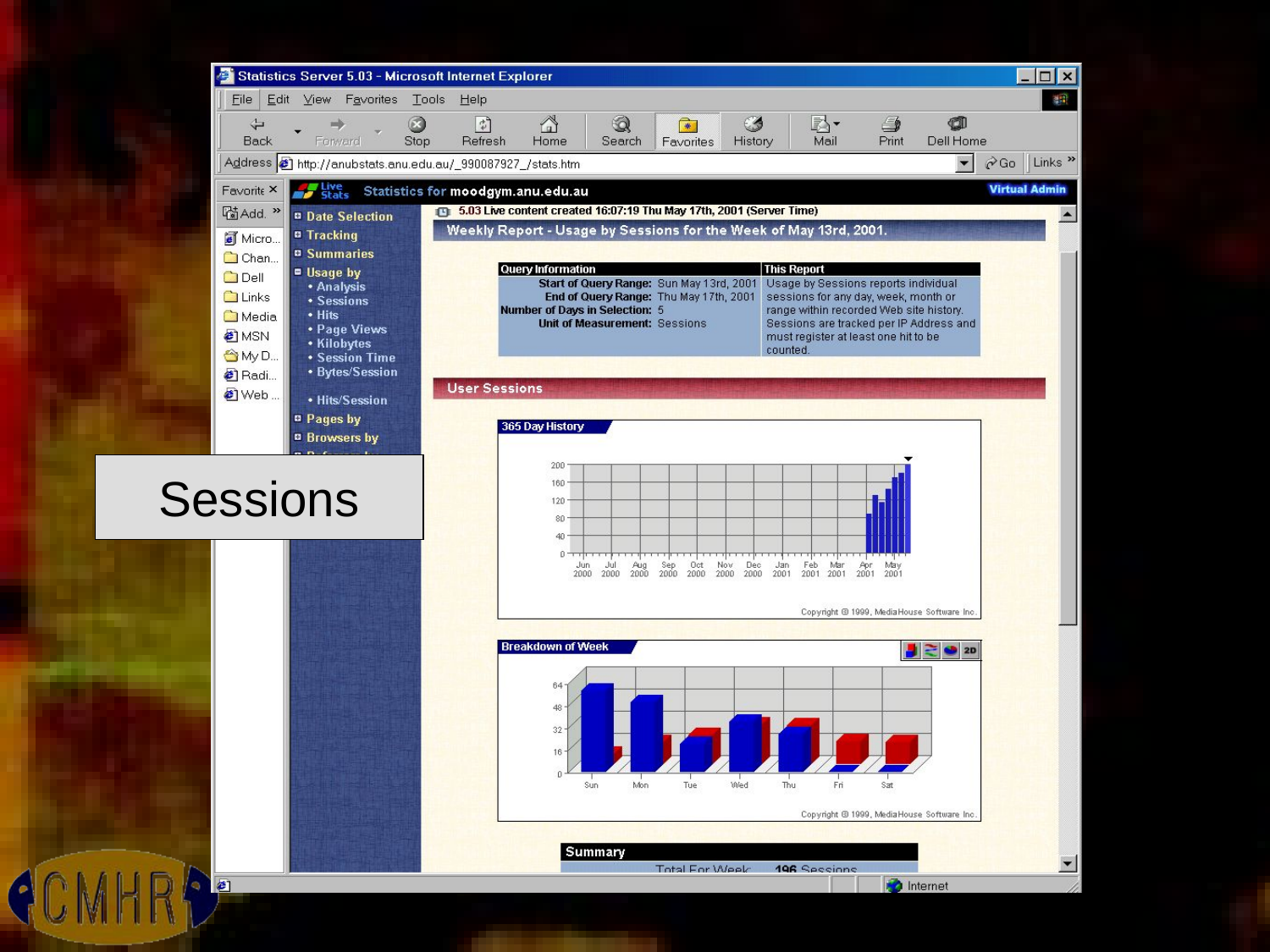

# Web stats summary
Sessions

## Slide 26
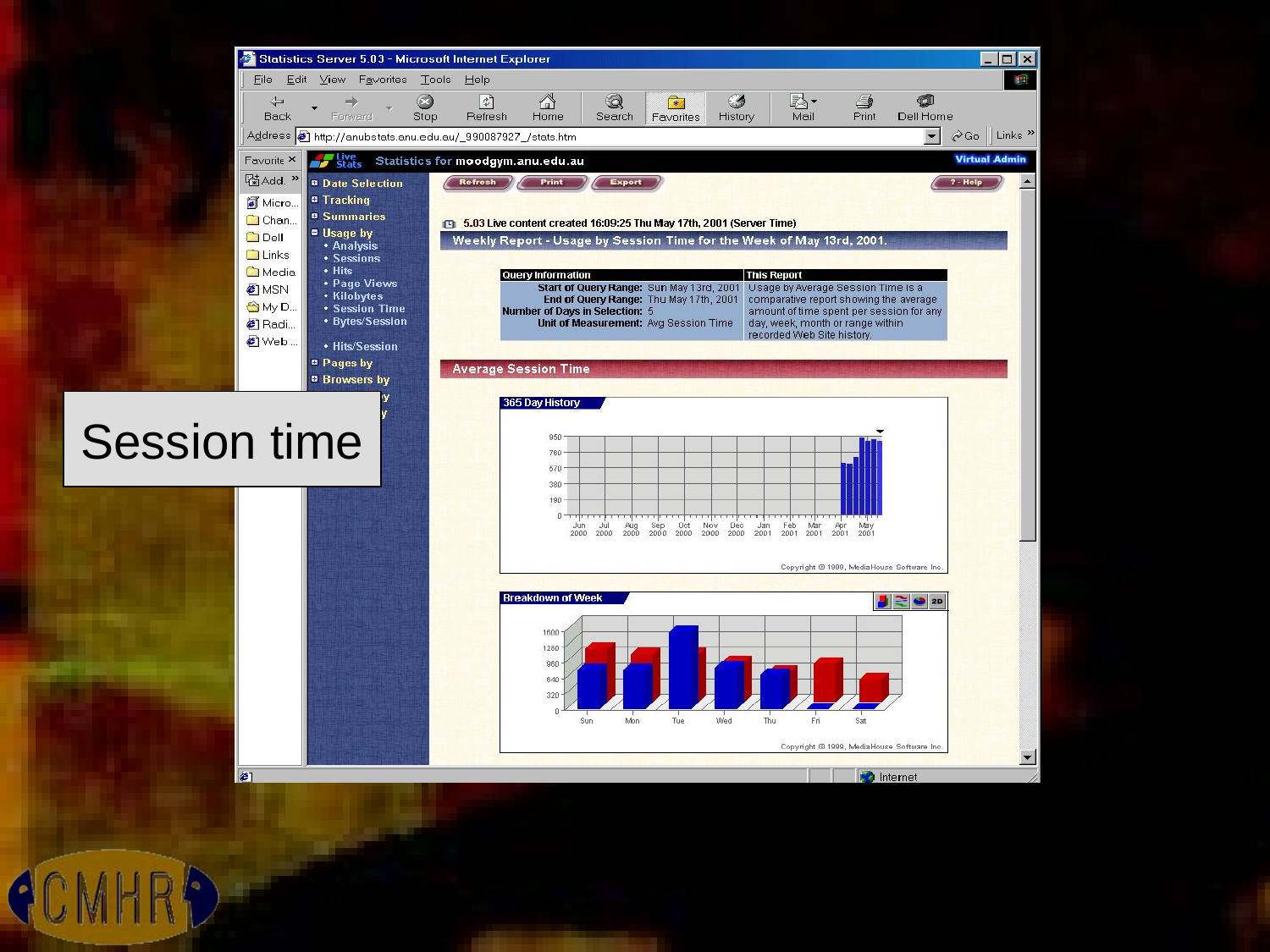

Session time

## Slide 27
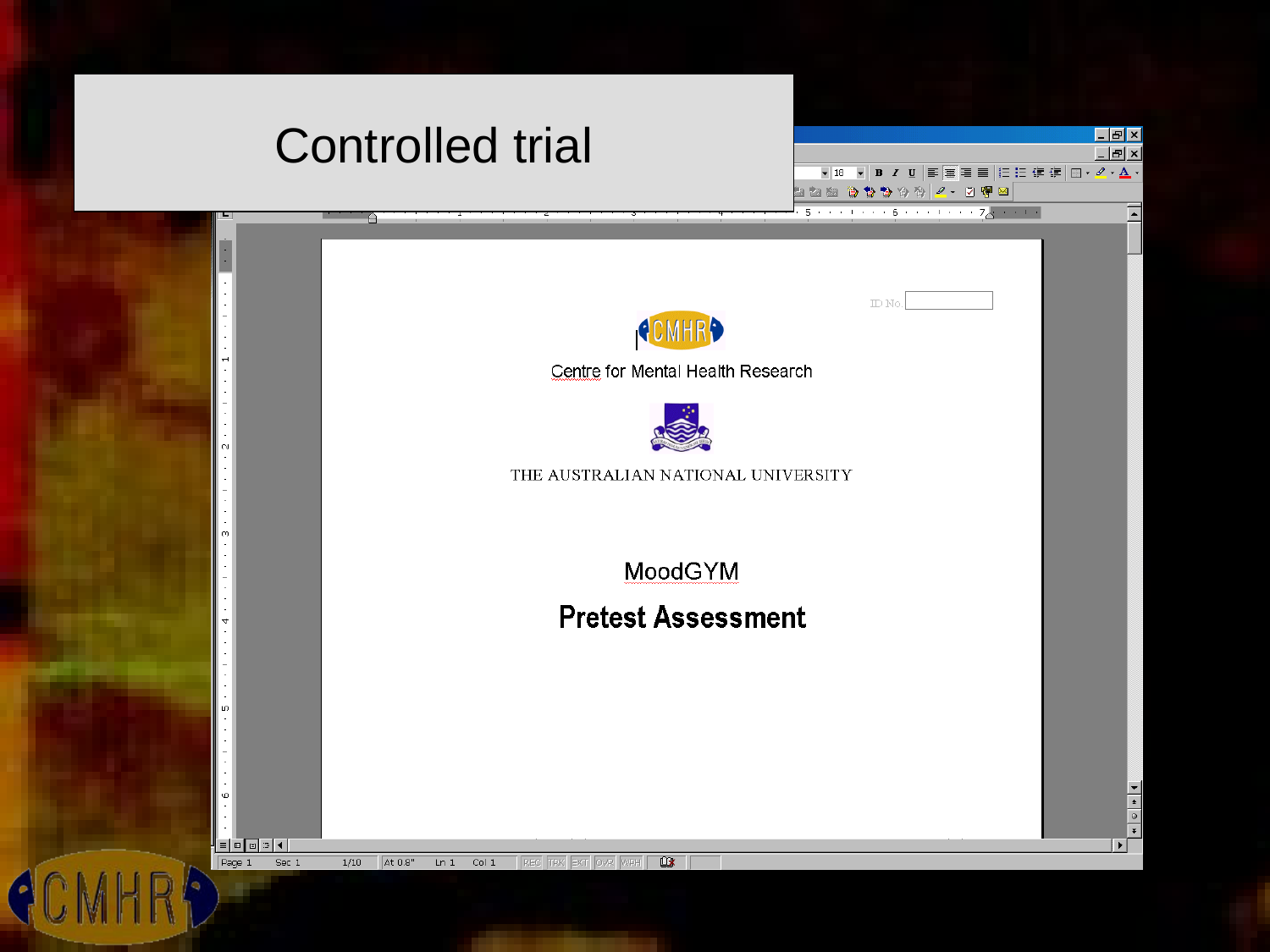

Controlled trial

## Slide 28
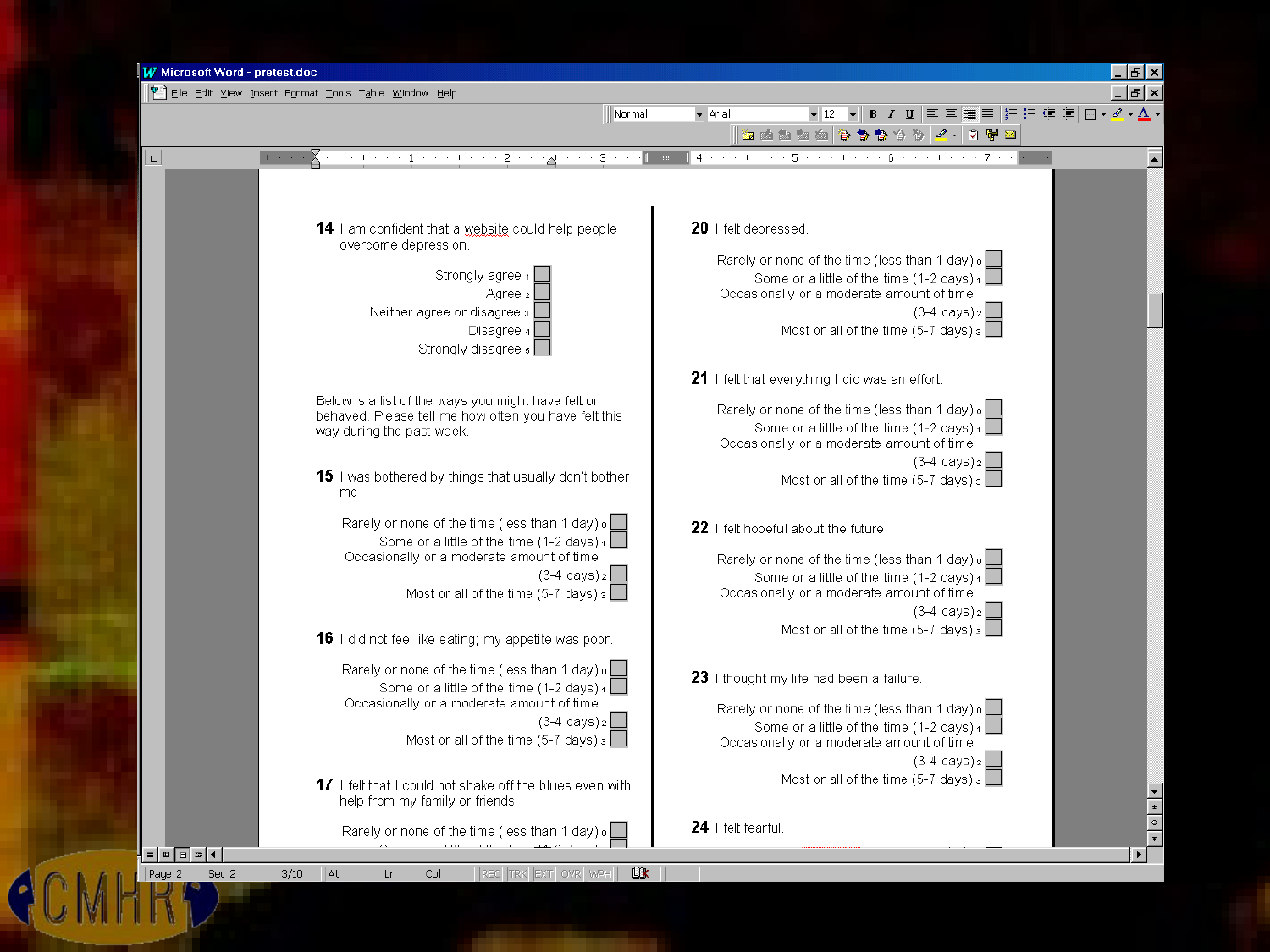

## Slide 29
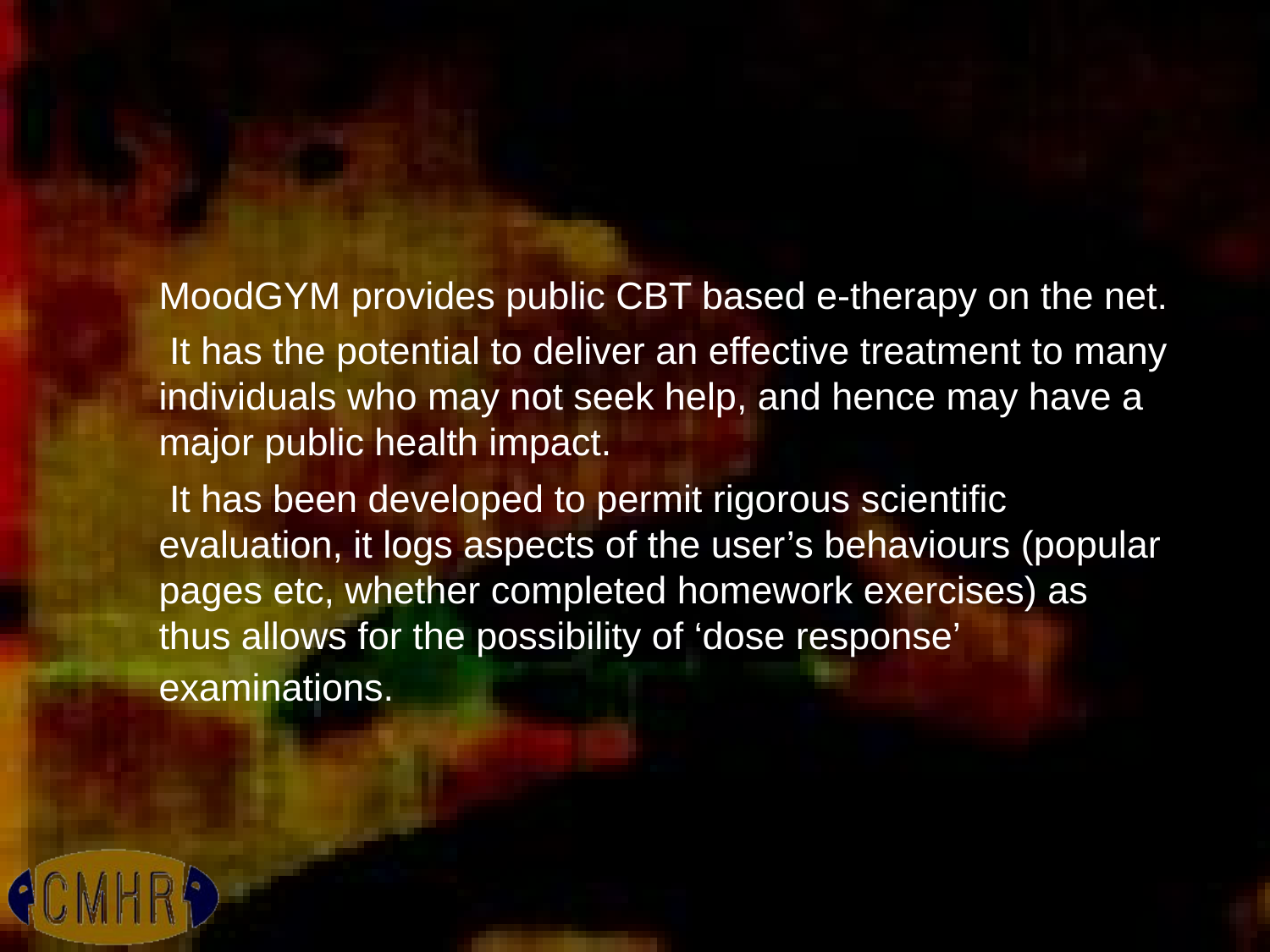

#
MoodGYM provides public CBT based e-therapy on the net.
 It has the potential to deliver an effective treatment to many individuals who may not seek help, and hence may have a major public health impact.
 It has been developed to permit rigorous scientific evaluation, it logs aspects of the user’s behaviours (popular pages etc, whether completed homework exercises) as thus allows for the possibility of ‘dose response’ examinations.

## Slide 30
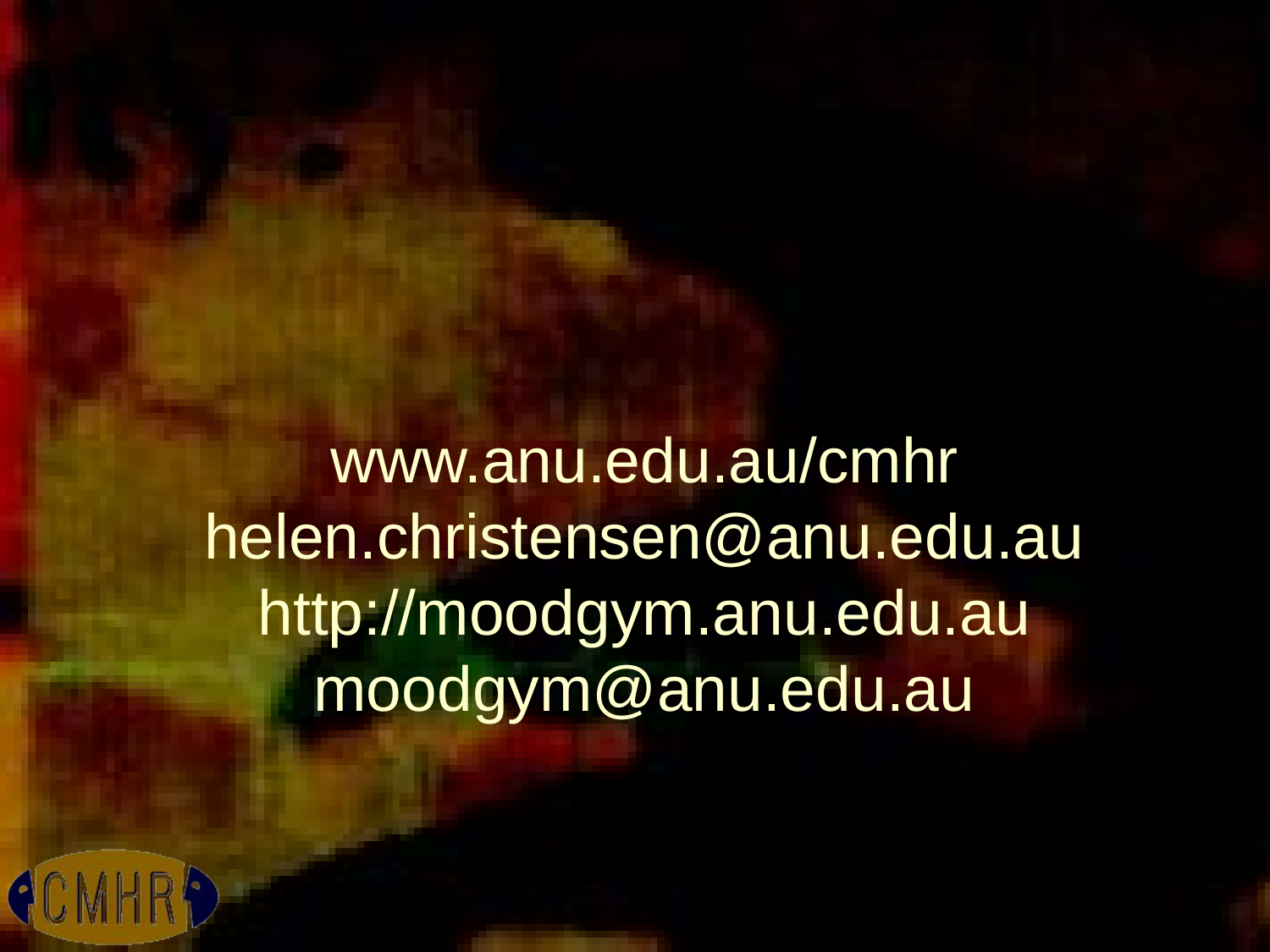

www.anu.edu.au/cmhr
helen.christensen@anu.edu.au http://moodgym.anu.edu.au
moodgym@anu.edu.au
